# Instruction on the Scientific Method Provides (Some) Protection Against Illusions of Causality

**DOI:** 10.1162/opmi_a_00141

**Published:** 2024-05-10

**Authors:** Julie Y. L. Chow, Micah B. Goldwater, Ben Colagiuri, Evan J. Livesey

**Affiliations:** School of Psychology, University of New South Wales, Sydney; School of Psychology, The University of Sydney, Sydney

**Keywords:** illusion of causality, contingency learning, base-rate instruction, outcome density, causal learning

## Abstract

People tend to overestimate the efficacy of an ineffective treatment when they experience the treatment and its supposed outcome co-occurring frequently. This is referred to as the *outcome density* effect. Here, we attempted to improve the accuracy of participants’ assessments of an ineffective treatment by instructing them about the scientific practice of comparing treatment effects against a relevant base-rate, i.e., when no treatment is delivered. The effect of these instructions was assessed in both a trial-by-trial contingency learning task, where cue administration was either decided by the participant (Experiments 1 & 2) or pre-determined by the experimenter (Experiment 3), as well as in summary format where all information was presented on a single screen (Experiment 4). Overall, we found two means by which base-rate instructions influence efficacy ratings for the ineffective treatment: 1) When information was presented sequentially, the benefit of base-rate instructions on illusory belief was mediated by reduced sampling of cue-present trials, and 2) When information was presented in summary format, we found a *direct* effect of base-rate instruction on reducing causal illusion. Together, these findings suggest that simple instructions on the scientific method were able to decrease participants’ (over-)weighting of cue-outcome coincidences when making causal judgements, as well as decrease their tendency to over-sample cue-present events. However, the effect of base-rate instructions on correcting illusory beliefs was incomplete, and participants still showed illusory causal judgements when the probability of the outcome occurring was high. Thus, simple textual information about assessing causal relationships is partially effective in influencing people’s judgements of treatment efficacy, suggesting an important role of scientific instruction in debiasing cognitive errors.

## INTRODUCTION

Advances in scientific methodology over the last 50 years have allowed medical science to achieve a level of evidential scrutiny that has not been possible at any other point in history. The advent and wide-spread adoption of the randomised controlled trial (RCT), for instance, has allowed the medical community to claim with some certainty that specific medications and treatments are genuinely effective, while others are not. The premise of much of this medical research is to randomise large samples of participants to receive the treatment or not and then compare the likelihood of a specific target outcome (e.g., illness, pain) across these groups. The difference in outcome likelihood for those treated and untreated can then be simultaneously calculated and used to provide an assessment of whether or not that particular treatment is efficacious and whether to use it in clinical practice.

However, whether due to lack of access to or knowledge of these techniques, most individuals do not call upon medical research to assess whether a treatment is likely to be effective for them. Instead, they often rely on evidence collected across many instances, potentially accumulating observation of their own experiences taking the particular treatment, the direct observation of others’ experiences with the treatment, or experiences relayed through conversation and various media. The opinion an individual forms is thus based on evidence that is experienced *sequentially*, non-scientifically, and not in the kind of summary form that lends itself to fast and accurate evaluation. As such, the types of ‘evidence’ that individuals base their decisions on is often very different from what medical researchers and the scientific community would consider of evidential value.

### Biased Contingency Learning and Illusory Beliefs

Researchers in cognition have for many years been interested in how and under what circumstances individuals can arrive at accurate judgments about causal relationships when they are exposed to cause-effect information in a sequential fashion. This is often referred to as *contingency learning*. Contingency in this case is typically defined using a metric that captures the change in the probability of the outcome when the potential cause (commonly referred to as the cue) is present versus absent. Allan’s (1980) Δp metric of contingency, for instance, is just a difference between the probability of the outcome when the cue is present, p(outcome | cause present), and the probability of the outcome when the cue is absent, p(outcome | cause absent). Participants exposed to positive contingencies and negative contingencies (as defined in terms of Δp), reliably rate the cue as generating or preventing the outcome respectively, and are generally sensitive to the strength of the contingency to which they are exposed. Thus, humans appear quite good at detecting genuine relationships when they do exist (Shanks & Dickinson, 1988).

However, individuals exposed to zero contingency—instances in which the p(outcome) is unaffected by the presence of the putative cause, i.e., Δp = 0—show a consistent error in their judgments. Faced with a null contingency of this type, participants’ ratings tend to be incorrectly positive and the strength of this rating tends to correlate with the overall probability of the outcome, such that the more the outcome occurs overall the stronger their causal ratings, despite there being no genuine contingency (see Table 1 for an example of zero contingency with high probability of the outcome occurring). The tendency to judge an ineffective cue as being causal is sometimes referred to as the *illusion of causality*, while the tendency for this to be stronger when the probability of the outcome is high is referred to as the *outcome density* (OD) effect. Both effects have been replicated widely in a variety of causal learning tasks where the potential cause is either the participant’s own actions (e.g., Alloy & Abramson, 1979; Blanco & Matute, 2015), or a cue presented within a fictitious causal context such as a medical treatment scenario (e.g., Blanco et al., 2013). The illusion of causality and OD effects have been observed with outcomes presented as discrete events (i.e., “patient recovers” versus “patient does not recover”) and with different distributions of continuous outcomes (Chow et al., 2019; Double et al., 2020). The influence of a high outcome probability can also be found in real-world health beliefs, where treatments that are not scientifically validated to be efficacious are commonly used for the treatment of illnesses that have a high rate of spontaneous remission akin to a high base-rate of recovery (e.g., *Echinacea* use and the common cold, Barrett et al., 2010; Karsch-Völk et al., 2014; see also Blanco & Matute 2019).

**Table 1.** Example of a contingency matrix with a binary cue and outcome, where Δp = 0, cue density = 0.50 (equal number of cue present and cue absent trials) and probability of the outcome = 0.80 (i.e., high outcome density). Letters in each cell refer to the unique trial type (e.g., a cells are trials where both cue and outcome are present), and the number in parentheses indicate the number of that particular trial type occurring. In this example, the outcome occurs frequently (32/40 trials), but just as often when the cue is present (16) and when it is absent (16).

	Outcome Present	Outcome Absent
Cue Present	*a* cell (16)	*b* cell (4)
Cue Absent	*c* cell (16)	*d* cell (4)

In essence, illusory causation and OD effects suggest that individuals either overestimate the likelihood of the outcome occurring in the presence of the putative cause relative to the likelihood that the outcome would occur anyway, even without the cue—that is, the base-rate of the outcome—or it suggests that people disregard the probability that the outcome will occur in the absence of the cause when forming causal beliefs. This type of zero-contingency learning error is further compounded by cases where the putative cause occurs very frequently, a phenomenon known as the *cue density* effect. A high probability of the cue being present increases the illusion of causality as it limits the opportunity for observers to evaluate the base-rate of the outcome. In passive zero-contingency tasks, where participants have no control over the cues, higher cue density elicits stronger illusory causation particularly when combined with high outcome density (Blanco et al., 2013; Kao & Wasserman, 1993). In *active* zero-contingency tasks, where participants can choose to activate a cue (e.g., to administer a fictitious treatment), participants tend to activate the cue more often than not, sometimes referred to as the positive testing strategy (Mandel & Lehman, 1998), which creates high cue density and leads them to perceive a stronger causal relationship (Blanco et al., 2011). This is important because it suggests that when faced with an ineffective cue and a frequent outcome, people tend to perceive a causal relationship where none exists (illusory causation and outcome density effect) *and* generate conditions that are suboptimal for assessing the causal relationship (cue density effect) by limiting their opportunities to estimate the base-rate in the absence of the cause.

Matute et al. (2011) argue that illusory causation is indicative of pervasive biases in causal learning that contribute to unfounded medical beliefs. Some of these beliefs are particularly controversial because they may lead to people making risky and potentially harmful decisions (Lim et al., 2011) and their prevalence is currently on the rise in western society (Steel et al., 2018). Illusory causation is also recognised as a contributor to the persuasive influence of pseudoscience across many contexts (Lilienfeld et al., 2014). It is therefore important to ask not just what causes these effects, but also how they can be ameliorated.

### The Role of Base-Rate Comparison in Causal Judgements

Blanco and Matute (2015) showed that relatively simple models of associative learning anticipate the occurrence of illusory causation and the OD effect. Applying associative models to these processes assumes that connections in memory between representations of the cause and the outcome serve as an intuitive basis for causal judgements. As such, they are analogous to a heuristics approach to causal reasoning in which people rely on memory without questioning whether there is a better way to determine cause and effect (Le Pelley et al., 2017; Thorwart & Livesey, 2016). Indeed, recent work have found that for many real-world pseudoscientific health beliefs, including the efficacy of complementary and alternative medicine, people’s beliefs are consistent with their estimation of the contingency between events (Chow et al., 2021). A corollary of this is that it should be possible to encourage other modes of thinking and that doing so should reduce the illusion of causality. In other words, it is not that people *cannot* make accurate judgments about causation and its absence, merely that under some conditions, we are not disposed to doing so. Supporting this idea, Matute and colleagues have shown that providing prior knowledge in the form of base-rate training, for instance, can reduce such effects. Blanco and Matute (2019, Experiment 1) gave participants pre-training on a contingency learning task to induce expectations of high outcome base-rate. They were then presented with an identical zero-contingency learning task and made a causal judgement at the end. These participants’ causal ratings were compared against participants who were given no pre-training. The researchers found that participants presented with a high outcome density scenario who were pre-trained showed significantly reduced causal illusions at test relative to participants in the control group, suggesting that base-rate knowledge can effectively reduce the illusion of causality even under conditions that typically encourage strong illusory beliefs (see also Barberia et al., 2013).

Blanco and Matute’s findings are promising, in that they provide some evidence that base-rate knowledge may be important for reducing false causal beliefs. Nevertheless, as a potential intervention strategy to promote evidence-based decision-making, such as correctly judging a bogus treatment to be ineffective, the implementation of this approach may prove time-consuming, and in some cases, impractical. Instead, we were interested in whether a simpler and more subtle strategy of instructing participants about *why* comparison with the base-rate might be important for assessing treatment efficacy would reduce causal illusions. We developed instructions to encourage the consideration of base-rates by briefly describing the logic behind a randomised controlled trial. We explained the rationale of a control condition and why a drug needs to produce an outcome at a greater rate than the control to attribute efficacy to the drug (Experiment 1) and provided some illustrative examples (Experiment 2). We assumed that the basic logic of a control condition in scientific and medical research is broadly familiar to participants, and so these instructions primarily served as a reminder to try to “think scientifically” when making causal attributions. The merit of this approach is that not only is it less resource-intensive than providing pre-training on the task, it is also a more ecologically valid way of prompting individuals to consider base-rate information when evaluating causal relationships.

### The Present Study

The goal of the present study was to determine the effect of simple base-rate instruction on illusory causation, and specifically the outcome density effect. In particular, we wanted to assess whether such a manipulation specifically operates by reducing people’s instincts to overvalue cue-outcome coincidences (where the cue and the outcome are both present, i.e., *a* cell trials) at the cost of evaluating the outcome in the *absence* of the cue (*c* cell trials). If this is true, causal ratings for the ineffective treatment should reduce more dramatically in participants given base-rate information when the outcome probability is high. However, if memory representations built from sequential learning of this sort are resistant to reasoning, it may be difficult to overcome these memory biases with simple instructions to reason about the base-rate. If this is the case, manipulations to outcome density might continue to produce strong illusory beliefs that are unaffected by instructional manipulations.

Another potential consequence of base-rate instruction, which might also serve as a mechanism for changing people’s assessment of cause and effect, is a change in participants’ willingness to administer the drug to patients (specifically, a reduction in the propensity to administer the treatment). Where such a reduction occurs, it will in turn reduce the cue density effect. Thus, even if simple base-rate instructions were not sufficient to override memory representations, it might still be an effective de-biasing tool by encouraging effective sampling of information (i.e., equal sampling of cue-present and cue-absent information).

In Experiments 1 & 2, we tested the hypothesis that instructions about the importance of base-rate comparison would reduce causal ratings relative to standard (no base-rate) instructions. We additionally tested whether base-rate instructions influenced the choice to administer treatment when participants had the opportunity to decide whether to administer treatment or no treatment on each trial.

## EXPERIMENT 1 & 2: ACTIVE SEQUENTIAL SAMPLING OF COVARIATION INFORMATION

This project was approved by the Human Research Ethics Committee (HREC) of the University of Sydney, approval number 2017/839.

The goal of Experiments 1 & 2 was to determine if the inclusion of base-rate instruction would decrease the illusion of causality relative to standard instructions. The effect of base-rate information on the probability of cue administration and causal judgements was tested using an active trial-by-trial contingency learning task where covariational information is presented sequentially and participants could choose whether or not to administer the cue on each trial. The use of an active version of the task allowed us to determine if there was any effect of these instructions on both causal ratings and participants’ choice behaviour during the training phase. This is important because—as described above—the illusion of causality is often associated with a tendency for the individual to choose to apply the potential cause more often than choosing to withhold it, leading to cue density effects that exacerbate illusory causation. Hence, if the instructional manipulations reduce this propensity to oversample cue-present trials and overvalue cue-outcome coincidences (sometimes referred to as an *a*-cell bias, Kao & Wasserman, 1993), this should result in more equal sampling of cue-present and cue-absent trials.

In this experiment, participants were presented with a fictitious scenario in which they were told they would play the role of a doctor and medical researcher assessing the efficacy of a new treatment for a disease, while treating patients suffering from the disease. At the beginning of the study, participants either received standard instructions that did not alert them to the benefits of using base-rate information for assessing treatment efficacy, or they received additional instructions about base-rate comparison. In Experiment 1, this additional base-rate instruction comprised a single screen of text in which the purpose of a control condition in the context of an RCT was described. They were not instructed to use this information themselves, merely told why base-rate information is potentially informative. In Experiment 2, the base-rate information was supplemented with three short vignettes of fictitious drug trials and the claims made by drug companies regarding the efficacy of the treatment. Participants were required to assess the validity of the claims made about the treatment described in each vignette. They were then given feedback about the validity of the claims and why they were or were not appropriate given the evidence. To distinguish between the two base-rate instruction conditions, we will refer to the single page instructions as ‘weak’ base-rate instruction, and the inclusion of the vignettes as ‘strong’ base-rate instruction. Although the interactive vignettes in Experiment 2 go beyond the simple single-page description of an RCT in Experiment 1, the vignettes were still quick and easy to implement and could readily be applied to real-world practice. One important benefit of the vignette approach is that it also serves as a manipulation check that participants not only understand the importance of the base-rate, but they are also able to use it appropriately to form causal attributions. Since the two experiments were virtually identical, with the exception of the content of the base-rate instructions, we report the methods and results of the two experiments together. Note however that the data for each experiment were analysed separately.

### Methods

#### Participants.

Experiments 1 and 2 were both run using Amazon’s Mechanical Turk (MTurk) recruitment service. The study was open to all MTurk workers in the United States. The experiments were run online and participants were paid USD3 for their participation. The task typically took around 15 minutes to complete. We recruited a total of 280 participants in Experiment 1 with one dataset failing to save, (remaining 279: *M*_*age*_ = 35.4, *SD* = 9.92, Male = 173, Female = 102, NA / Choose not to say = 4), and 290 participants in Experiment 2 (*M*_*age*_ = 16.7, *SD* = 9.14, Male = 170, Female = 114, Other = 1, NA / Choose not to say = 5). We excluded participant data on a number of basic compliance checks. First, participants were given a series of four simple questions testing for basic retention and understanding of the instructions. If they answered any of these questions incorrectly then the instructions were repeated. Participants’ data were removed if they failed this check more than twice. Participants were also asked at the end of the experiment to answer honestly if they had written anything down during the experiment, those who admitted to writing down information were removed from analyses. In Experiment 1, we additionally included a ‘bot check’ at the end of the experiment, where participants were asked to select Option 1 from four possible options. This generic bot check was replaced with a simple memory question in Experiment 2, where participants were asked to recall the name of the drug used as treatment in the experiment (choosing between 4 possible options); participants who failed to select Option 1 in the bot check (Experiment 1) and those who failed to recall the correct drug name (Experiment 2) were removed from analyses. A breakdown of the number of excluded datasets by exclusion criteria for all experiments reported in this paper can be found in Table S1 in Supplementary Materials.

Allocation of participants to condition was random, and we aimed to collect enough data to have usable data sets from approximately 60 participants in each group. This led to a final sample of 245 for Experiment 1 (low OD standard instruction *n* = 59, low OD weak instruction *n* = 60, high OD standard instruction *n* = 62, high OD weak instruction *n* = 64) and 258 for Experiment 2 (low OD standard instruction *n* = 71, low OD strong instruction *n* = 61, high OD standard instruction *n* = 64, high OD strong instruction *n* = 62).

#### Design.

Both experiments used a between-subjects design crossing 2 levels of outcome density—low (infrequent, 20% probability of recovery) versus high (frequent, 80% probability of recovery)—with 2 levels of instruction—standard versus added base-rate information—resulting in four groups in total. Participants were presented with a contingency learning task with 40 ‘patients’ (i.e., 40 learning trials) in total, and for each had the choice to administer the fictitious treatment or to give no treatment. Participants made this choice, gave a rating on the patient’s chances of recovery, and then observed the consequences (presented as a binary outcome of “Patient has recovered” or “Patient has not recovered”). In this task, p(outcome | cue) is a measure of the proportion of trials where patients were given treatment (Cloveritol) and subsequently recover from illness, and p(outcome | no cue) is the proportion of trials where patients were given no treatment and recover from illness.

At the end of 40 trials, they gave a causal rating about the efficacy of the treatment. Efficacy ratings taken at the completion of the learning task constituted the key indicator of (illusory) causal judgment. We also measured the probability of choosing to administer the treatment by taking the number of trials on which they chose treatment divided by the total number of trials (i.e., 40) in the contingency learning task.

#### Apparatus & Stimuli.

**Figure 1.**
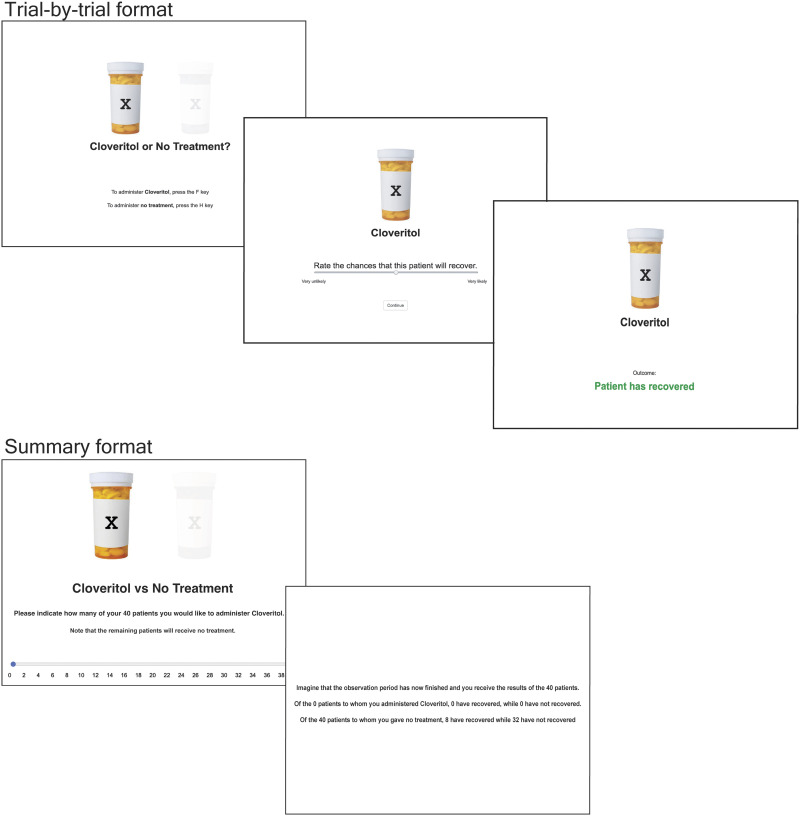
Example of the sequence of events within a single trial in the trial-by-trial format of the contingency learning task used in Experiments 1 & 2 (top panel), and in summary format in Experiment 4 (bottom panel). Experiment 3 implemented a version of the trial-by-trial contingency task which excluded the opportunity to select whether to administer Cloveritol or No Treatment.

The task was programmed using custom scripts written based on plugins available in the JSPsych library (de Leeuw, 2015). During the contingency learning task, the administration of the treatment cue was represented graphically by a pill bottle labelled X, with the name “Cloveritol” written beneath. Administration of no treatment was depicted by a faint pill bottle outline, and the words “No treatment” written underneath. The image and text for the (chosen) cue or no treatment were always present on the screen on any given trial. Prediction rating scales and patient outcomes were presented beneath the image and text (see Figure 1).

#### Procedure.

Participants first read generic instructions informing them that they were to play the role of a doctor and medical researcher in a hypothetical scenario. They were instructed that they were treating ill patients with a new experimental drug, Cloveritol, and that their task was to assess how effective Cloveritol was at treating the illness while treating a series of patients, presented one at a time. Participants in the standard instruction condition in both experiments were then provided with instructions on how to complete the experiment. Participants in the added base-rate instruction conditions, on the other hand, were given additional information about the relevance of comparing the recovery rate of patients who receive the drug to the recovery rate of patients who do not receive treatment. In Experiment 1, we used a relatively subtle one-page version of the following instructions:

*“New drugs are normally tested using a clinical trial. Clinical trials typically involve giving the drug to some patients but giving other patients no treatment. If the chances of recovery are higher for the patients that were given the drug, it suggests that the drug has some effect on recovery. If the chances of recovery are the same for patients given the drug and patients given no treatment, it suggests that the drug has no effect on recovery.”*

In Experiment 2, we added additional interactive elements to the instructions. In addition to the instructions above, participants read three vignettes of statements made by a fictitious drug company and were asked to assess whether the evidence presented in these statements was good evidence that the company’s claims about drug efficacy were true. They were given feedback after each vignette; feedback on each of the three vignettes also included a brief explanation as to *why* the evidence supported or did not support the company’s claims by highlighting the base-rate information (e.g., A higher rate of recovery was observed in those people who took the drug than those who did not). The three vignettes and their subsequent feedback are provided in Materials S1 of the Supplementary Materials. The instructions on base-rate comparison in the weak and strong instruction conditions were the only differences between the standard instructions and the added base-rate instruction conditions. Participants were then presented with identical instructions on how to complete the contingency learning task. All participants were told that they would be a total of 40 trials (each representing a different patient), and they would be required to use the information obtained from these trials to make a judgement about the effectiveness of Cloveritol.

During the contingency learning task, participants were first provided a choice between administering Cloveritol or administering no treatment to the patient. The graphical depiction of this choice is shown in the first image of the top panel of Figure 1. On choosing one of these options, participants then rated the likelihood that the patient would recover using a linear analogue scale from ‘Very unlikely’ to ‘Very likely’. They then clicked on the Continue button when they were ready to observe the patient’s outcome. Patient outcomes were presented in a binary fashion in text: Patient has recovered / Patient has not recovered. At the completion of 40 trials of the contingency learning task, participants were asked to rate how effective the drug was at treating the disease, relative to no treatment. This efficacy rating was made on a scale from −100 (Effectively worsens the disease) to 100 (Effectively treats the disease) with a midpoint of 0 (Completely ineffective).

### Results

We were primarily interested in whether base-rate instructions presented at the start of the experiment had an influence on two key dependent variables: efficacy ratings for the treatment provided at the end of the experiment and probability of administering the treatment during the learning task. We additionally report participants’ *experienced* treatment-outcome contingency. Although we collected prediction ratings on each trial, this was not a critical measure for our research aims, and were primarily used to keep participants focused on the task during the learning phase. These results are presented in Supplementary Materials. The full dataset for all experiments reported here is publicly available at the Open Science Framework, and can be accessed at https://osf.io/9wckv/.

With the exception of the mediation analysis reported at the end of this section comparing the effect of instructions on efficacy ratings (controlling for the probability of treatment administration), results for Experiment 1 and Experiment 2 were analysed separately. Primary analyses were conducted with both Null Hypothesis Significance Testing (NHST) and Bayes Factor Analysis. Bayes Factors for main effects are reported as a likelihood ratio of the alternative model relative to the null model. Where there are more than two factors in the model, we reported the Bayes Factor Inclusion (BF_incl_) across matched models (Rouder et al., 2017), which provides an estimate for the evidence for the effect to equivalent models stripped of the effect. Bayesian analyses were conducted using the BayesFactor package in R (v0.9.12-4.4, Morey & Rouder, 2022). By default, the package utilizes the Jeffreys-Zellner-Siow (JZS) prior for Bayes factors. In this model, standardized treatment effect, *δ* is assumed to be 0 under the null hypothesis. Conversely, under the alternative hypothesis, the prior distribution for *δ* follows a Cauchy distribution with scale parameter, *r* = .5 for fixed effects in an Analysis of Variance. A detailed description of the default prior computation and implementation can be found in Rouder et al. (2012) or in the BayesFactor R package documentation (Morey & Rouder, 2022).

#### Experienced Contingency.

**Figure 2.**
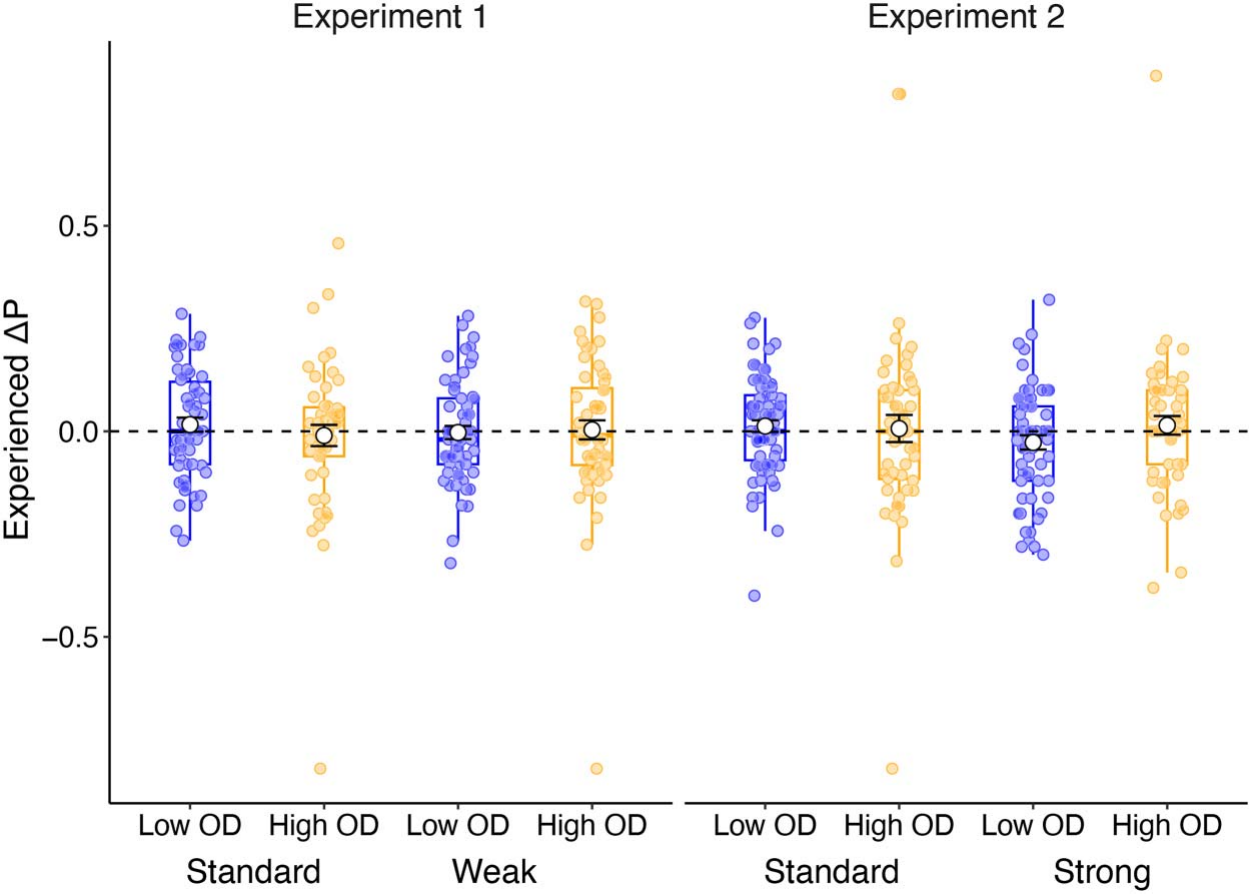
Average experienced Δp (white circles illustrate the mean± standard error) as a function of instructional condition (Standard vs Base-rate instructions) and outcome density (OD) condition (Low vs High), separated by Experiment. Each coloured circle represents an individual participant data point plotted over boxplots illustrating the inter-quartile range and median for each condition.

We will first present the relevant hypothesis followed by a description of the statistical analysis approach before presenting the results from both experiments. Although the task was designed to have zero contingency between treatment and recovery, we did not have full control over when the treatment cue was present or absent due to participants choosing on each trial whether to administer the drug or not, and thus small deviations from zero contingency were inevitable. Thus, for each participant, we calculated their *experienced* treatment-outcome contingency (quantified as Δp) by taking the difference in the probability of the outcome on treatment trials and no treatment trials, p(outcome | cue) − p(outcome | no cue). Participants who exclusively sampled treatment-present or exclusively sampled treatment-absent trials were not included in this analysis because it is impossible to calculate Δp without sampling *both* types of trial. After excluding participants who failed to sample both trial types, we were left with 223 valid datasets in Experiment 1 and 238 in Experiment 2. Overall, average experienced Δp was close to the veridical value of zero and did not differ substantially between conditions (see Figure 2). See Table S2 in Supplementary Materials for a summary of average experienced Δp for participants in each of the four groups in Experiment 1 and 2 separately.

Here, we report the correlation between participants’ experienced Δp and their efficacy ratings at the end of the experiment, to determine whether causal judgements were influenced by their experienced covariation information. We additionally ran an Analysis of Variance (ANOVA) to compare group-level experienced Δp as a function of OD condition and instruction (standard vs base-rate instruction).

##### Experiment 1.

We predicted and subsequently found experienced contingencies to be positively correlated with participants’ judgements of treatment efficacy, *Pearson’s r* = .204, *p* = .002, BF_10_ = 9.32, however we did not find participants’ experienced contingency to be significantly different as a function of instructions, *F*(1, 221) = .017, *p* = .897, *η*_p_^2^ < .001, BF_Incl_ = .148, or OD condition, *F*(1, 221) = .234, *p* = .629, *η*_p_^2^ = .001, BF_Incl_ = .161. We also found no interaction between the two factors, *F*(1, 221) = .637, *p* = .426, *η*_p_^2^ = .003, BF_Incl_ = .258.

##### Experiment 2.

Experienced contingency was again positively correlated with participants’ judgements of treatment efficacy, *Pearson’s r* = .285, *p* < .001, BF_10_ = 1808. As in Experiment 1, we did not find experienced contingency to be significantly different as a function of instructions, *F*(1, 236) = .535, *p* = .465, *η*_p_^2^ = .002, BF_Incl_ = .192, or OD condition, *F*(1, 236) = .683, *p* = .409, *η*_p_^2^ = .003, BF_Incl_ = .187, nor was there an interaction between the two factors, *F*(1, 236) = 1.21, *p* = .273, *η*_p_^2^ = .005, BF_Incl_ = .347. Mean Δp in all four groups were again close to zero.

#### Probability of Treatment Administration.

**Figure 3.**
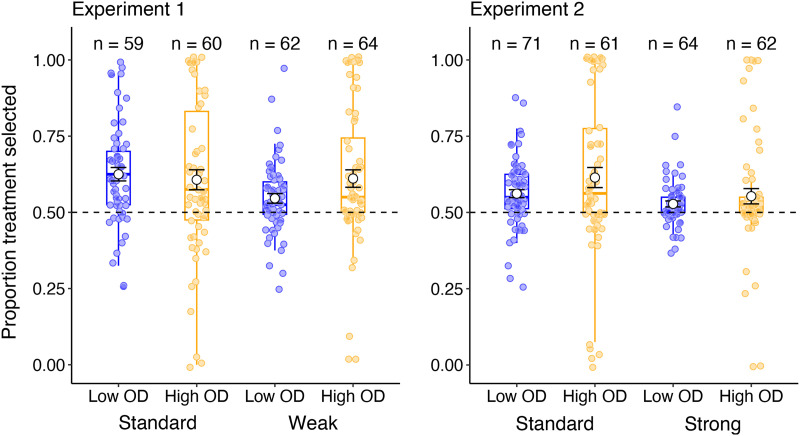
Average proportion of trials (± standard error) where the treatment cue was selected throughout the training phase, as a function of outcome density (OD) and instructions in Experiment 1 (left panel) and Experiment 2 (right panel). Values above 0.5 indicate an overall preference for sampling the treatment cue, values below 0.5 indicate a preference for no treatment, and values around 0.5 indicate an unbiased sampling of treatment and no treatment trials.

In order to determine if the instructions presented influenced the sampling of treatment and no treatment trials during training, we analysed the overall proportion of treatment-selected trials as a function of OD and instruction condition using an Analysis of Variance (ANOVA). We hypothesised that participants who received base-rate instructions were less likely to sample treatment trials compared to standard instructions, and more likely to sample both treatment and no treatment trials at an equivalent rate (i.e., probability of cue administration = 0.5). The balanced sampling strategy (50/50 cue and no cue trials) provides participants with the best opportunity to compare the probability of the outcome occurring with and without the putative cause present, and should therefore assist in forming accurate causal judgements. We additionally used a Student’s *t*-test to compare the average proportion of cue selection in each group to a value of 0.5 (equal sampling) to determine whether there is any evidence of biased sampling of cue information. These results are illustrated in Figure 3, with the sample size of each condition included above the relevant condition as a reminder.

##### Experiment 1.

ANOVA comparing OD and instructions on probability of treatment administration revealed no main effect of instruction or OD, largest *F* = 2.10. There was also no significant interaction between the two factors, *F*(1, 241) = 2.58, *p* = .110, *η*_p_^2^ = .011, BF_Incl_ = .593. The proportion of cue trials selected was statistically greater than 0.5 in both the standard group, *t*(118) = 6.11, *p* < .001, *d* = .560, BF_10_ = 2.17e+5, and the weak instruction group, *t*(125) = 5.03, *p* < .001, *d* = .448, BF_10_ = 2354.

##### Experiment 2.

Experiment 2 showed a significant main effect of instructions on probability of treatment administration, *F*(1, 254) = 4.63, *p* = .032, *η*_p_^2^ = .018, BF_Incl_ = 1.10, suggesting that the additional vignettes may be more beneficial in reducing over-sampling of cue trials. The Bayes Factor however suggests that this finding may not be conclusive. There was no main effect of OD, *F*(1, 254) = 3.13, *p* = .078, *η*_p_^2^ = .012, BF_Incl_ = .599, and no significant interaction between the two factors, *F* < 1. The proportion of cue trials selected was statistically greater than 0.5 in both the standard group, *t*(131) = 5.45, *p* < .001, *d* = .475, BF_10_ = 8966, and the strong instruction group, *t*(125) = 4.03, *p* < .001, *d* = .359, BF_10_ = 7.13.

Across the two experiments, there is no conclusive evidence that the presentation of base-rate information in the instructions reduced sampling of cue trials during training. Thus, any benefit of base-rate instruction was not sufficient to overcome participants’ tendency to over-sample.

#### Efficacy Ratings.

**Figure 4.**
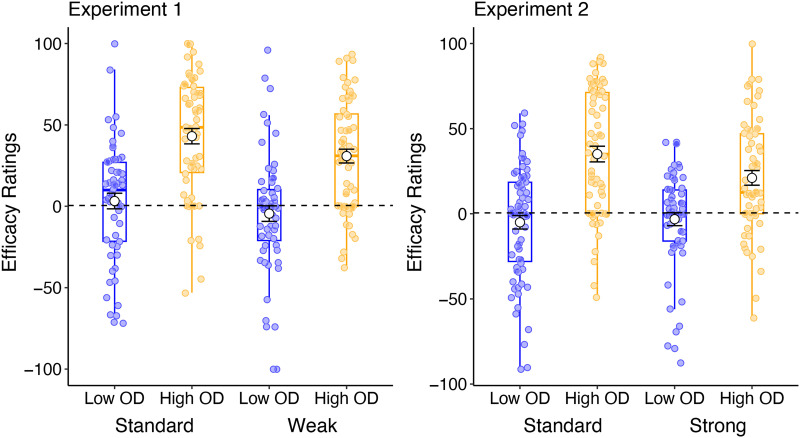
Average causal ratings provided at test (white circle illustrate the mean ± standard error) on the efficacy of the drug in treating the illness relative to no treatment, as a function of outcome density (Low OD vs High OD) and instruction (Standard vs Base-rate instructions) in Experiment 1 (left panel) and Experiment 2 (right panel). Efficacy ratings are measured on a scale from −100 (Effectively worsens the disease) to 100 (Effectively treats the disease) with a midpoint of 0 (Completely ineffective).

Efficacy rating for the treatment relative to no treatment was the critical dependent variable for observing any effect of outcome density on causal belief. We were interested in determining whether the OD effect—indexed by greater causal ratings for participants in the High OD condition relative to the Low OD condition—was influenced by the information presented in the instructions. We first ran an ANOVA with efficacy ratings as the dependent variable, and instructions and OD as between-subjects factors. As alluded to in the introduction, we expect that any effect of base-rate information on causal ratings will be most evident under High OD conditions, where illusory causation is typically strongest. To determine this, we compared the effect of base-rate instructions on causal ratings for participants in the High OD condition only. A reduction in causal ratings for participants given base-rate instructions compared to the standard instruction group would suggest that the additional base-rate instructions were effective in influencing the way participants evaluate the evidence for causal association before making a judgment. These results are illustrated in Figure 4.

Average causal ratings were also compared to a value of zero (normative rating for a non-contingent cue) to determine if base-rate instructions were effective at completely removing illusory causal beliefs in the treatment, and finally, we report the results from a linear regression to determine whether probability of treatment administration was a significant predictor of causal ratings over and above participants’ experienced contingency.

##### Experiment 1.

ANOVA comparing the effects of OD and instruction on causal ratings showed an overall main effect of OD, *F*(1, 241) = 65.9, *p* < .001, *η*_p_^2^ = .215, BF_Incl_ = 2.14e+11, indicative of an outcome density effect. There was also a main effect of instructions, *F*(1, 241) = 4.64, *p* = .032, *η*_p_^2^ = .019, BF_Incl_ = 1.27, but no interaction between the two factors, *F*(1, 241) = .236, *p* = .627, *η*_p_^2^ < .001, BF_Incl_ = .205. Comparing only the effects of instruction in the High OD group where the benefit of base-rate instructions was expected to be greatest (Bonferroni corrected alpha = .02), we found no difference between the two groups, *F*(1, 124) = 3.74, *p* = .055, *η*_p_^2^ = .029, BF_10_ = 1.02. Similarly, no difference was found in the Low OD groups, *F*(1, 117) = 1.30, *p* = .257, *η*_p_^2^ = .011, BF_10_ = .350.

A *t*-test comparing mean efficacy ratings to the normative value of zero for participants in the High OD condition showed that average ratings were still significantly above zero for both participants in the weak instruction group, *t*(63) = 7.29, *p* < .001, *d* = .911, BF_10_ = 1.69e+7, as well as the standard instruction group, *t*(61) = 9.15, *p* < .001, *d* = 1.16, BF_10_ = 1.69e+10. Finally, we ran a linear regression on the effect of probability of treatment administration on efficacy ratings controlling for participants’ experienced contingencies. Here, we found that over and above the effect of experienced Δp, the probability of treatment administration was a significant predictor of participants’ causal judgements at test, Δ*R*^2^ = .094, Δ*F*(2, 224) = 11.5, *β* = .251, *t*(224) = 3.58, *p* < .001, BF_10_ = 70.1.

##### Experiment 2.

ANOVA results revealed a main effect of OD, *F*(1, 254) = 58.8, *p* < .001, *η*_p_^2^ = .188, BF_Incl_ = 2.08e+11, and a marginal interaction between OD and instructions, *F*(1, 254) = 3.56, *p* = .060, *η*_p_^2^ = .014, BF_Incl_ = 0.227. There was no main effect of instructions on efficacy ratings, *F*(1, 254) = 2.10, *p* = .148, *η*_p_^2^ = .008, BF_Incl_ = 1.22. Looking only at the High OD group, we found a marginal difference between the two instructions group, *F*(1, 124) = 4.93, *p* = .028, *η*_p_^2^ = .038, BF_Incl_ = 1.74, but the Bayes Factor suggests only anecdotal evidence. There was no significant effect of instructions on efficacy ratings in the Low OD group, *F* < 1. *T*-tests again showed that efficacy ratings were significantly greater than zero for both participants in the High OD condition who received strong base-rate instructions, *t*(61) = 4.90, *p* < .001, *d* = .622, BF_10_ = 2367, as well as those who received standard instructions, *t*(63) = 7.63, *p* < .001, *d* = .953, BF_10_ = 6.26e+7. These findings suggest that simple base-rate instruction alone was not sufficient to completely overcome biases in causal judgement. Linear regression on the effect of probability of treatment administration on efficacy ratings controlling for experienced contingency again revealed a significant effect of cue-sampling behaviour on causal judgements above and beyond experienced Δp, Δ*R*^2^ = .125, Δ*F*(2, 239) = 16.9, *β* = .230, *t*(239) = 3.42, *p* < .001, BF_10_ = 40.3. These results support previous findings that the extent to which people over-sample cue-present trials influence the strength of illusory causation.

#### Mediation Analysis.

To consider the effects of treatment selection together with efficacy ratings, we conducted a mediation analysis on the effect of base-rate instructions on causal ratings, with the probability of treatment administration as a mediator and controlling for Experiment as a factor. This was done using the PROCESS function in R (Hayes, 2017). Variance estimation was computed using non-parametric bootstrap (number of samples = 1000). For greater statistical power, we collapse data across both Experiments 1 & 2 and included Experiment as a covariate in the model in this analysis. Note that the only difference between Experiments 1 & 2 was the extent of the instructions on randomised controlled trials—single page description about RCTs in Experiment 1, and a short practice comparing between treatment and no treatment outcomes using vignettes in Experiment 2. Critically we were interested in whether the inclusion of base-rate instructions significantly reduced causal ratings when the probability of treatment administration was controlled.

**Figure 5.**
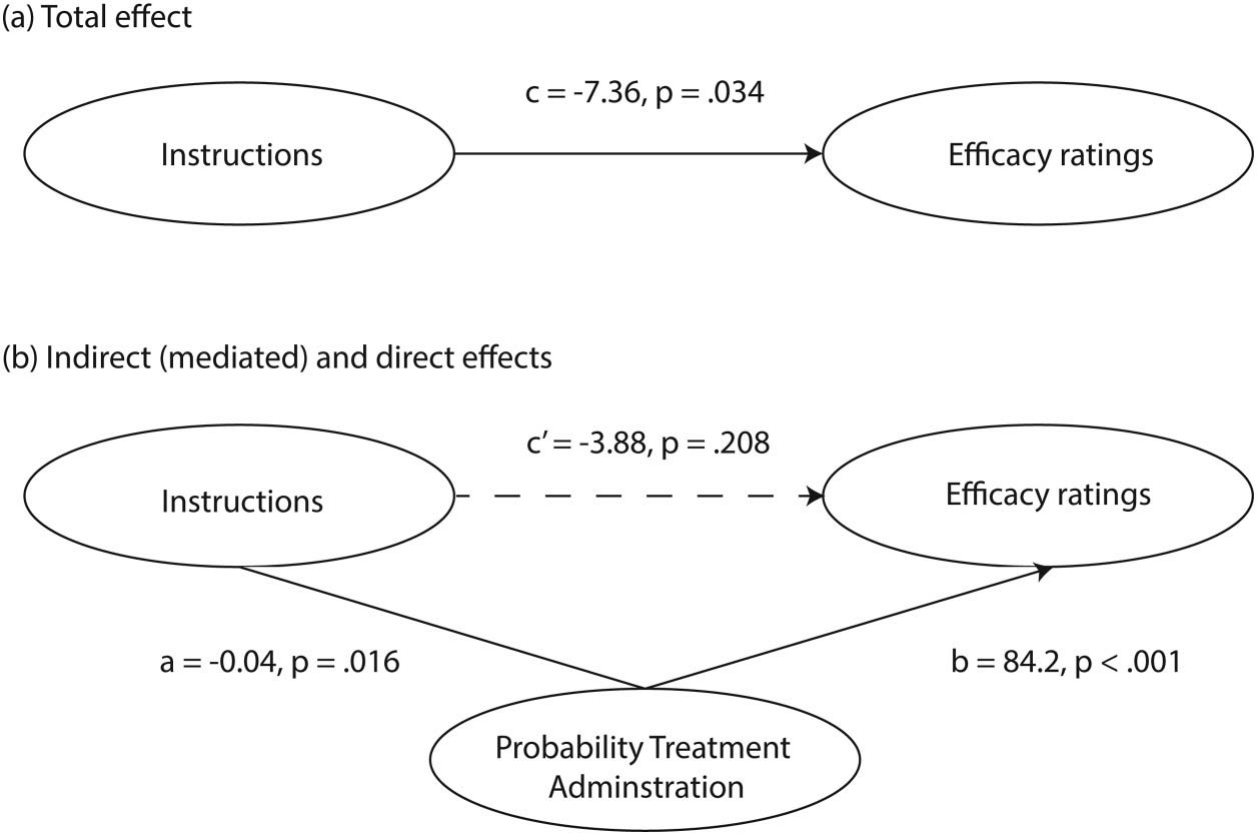
Mediational structure depicting the relationship between instruction, probability of treatment administration and efficacy ratings (with Experiment as a covariate). *Note*. Instructional manipulation was dummy coded with 0 for standard instructions and 1 for base-rate instructions. (a) The total effect of instruction on efficacy ratings is significant, with negative estimate (denoted as *c*) suggesting a decrease in the magnitude of the relationship as we move from the standard to the base-rate instruction group. (b) Overall indirect effect of instructions on efficacy ratings through probability of treatment administration can be broken down into two pathways: *a* denoting the effect of instructions on treatment administration, and b the effect of treatment administration on efficacy ratings. Once probability of treatment administration is controlled, there was no direct effect of instructions on efficacy ratings, *c*′.

Based on the mediational structure hypothesis, the total effect (Figure 5a) of instructions on efficacy ratings is composed of two pathways, one direct effect and one indirect effect through probability of administration (Figure 5b). The analysis revealed a significant total effect of instructions on efficacy ratings, unstandardized coefficient, *c* = −7.36, *p* = .035, 95% CI ranged from −14.2 to −.51. The negative unstandardised estimate suggests that the addition of the base-rate instructions (standard condition coded as 0; additional base-rate coded as 1) was associated with a reduction in efficacy ratings. There was also a significant indirect effect of instructions on efficacy ratings via probability of administration, *a* * *b* = −3.48, 95% CI [−.16, −0.18]. However the direct effect of instructions was not significant once probability of treatment administration was controlled for, *c*′ = −3.88, *p* = .225, 95% CI [−10.2, 2.40]. These findings suggest that any effect of instructions on judgements of treatment efficacy can be explained solely by changes in the probability of administering the treatment during training.

Results of Experiments 1 & 2 suggest some influence of base-rate instructions on the illusion of causality. This effect was found to be meaningfully predicted by the probability of treatment administration over and above any differences in the participant’s experienced cue-outcome contingency. Although we did not find a significant effect of instructions on cue administration in the individual experiment results, this is likely due to the individual experiments being underpowered to detect the subtle effect of base-rate instruction. Results from the mediation analysis which combines data across the two experiments on the other hand, showed a significant effect of base-rate instructions on causal ratings, and this relationship was mediated by the probability of treatment administration. That is, participants given base-rate instructions had a lower overall probability of cue administration compared to those given standard instructions and this resulted in a reduction in illusory causal beliefs.

In Experiments 3 & 4, we further interrogate this relationship between base-rate instruction, cue administration and causal judgements. In Experiment 3, we conducted a passive version of the task reported in Experiments 1 & 2, where participants were not given the choice to administer the treatment or no treatment, but were told whether each patient received Cloveritol or no treatment. This allowed us to determine whether there was any effect of base-rate instruction on the *a*-cell bias in causal judgement, when cue administration was controlled. All other aspects of the task were identical to the previous experiments. For completeness, we included both base-rate instruction conditions from Experiments 1 & 2 in a single design and compared the weak base-rate instructions in Experiment 1 and the strong base-rate instructions in Experiment 2 to the standard instruction group. A similar study design was employed in Experiment 4, where we were interested in whether the effect of the instructional manipulation on cue administration and illusory causation would still be present when cue-outcome information was presented in summary form.

## EXPERIMENT 3: PASSIVE CONTINGENCY TASK WITH SEQUENTIAL PRESENTATION OF INFORMATION

In Experiment 3, we were interested in whether base-rate instructions had any *direct* influence on efficacy ratings when cue administration was controlled by the experimenter rather than the participant. In this experiment, we presented participants with a *passive* version of the contingency learning task, where participants had no choice over the activation of the cue, and were instead told at the start of each trial whether the cue was present (i.e., patient given treatment) or absent (patient given no treatment). This passive version of the experiment provides a more empirical assessment of the direct relationship between instructions and efficacy ratings assessed in the mediation analysis. We also directly compared the effects of weak and strong base-rate instructions in this experiment to determine whether additional efforts to engage participants in appropriate base-rate reasoning (and the opportunity to receive feedback about it) might lead to greater reduction in illusory beliefs in the experiment.

All participants were presented with 40 trials in total, however in an attempt to match the average number of cue administration in Experiments 1 & 2, 25 of the 40 trials (62.5%) were treatment trials and the remaining 15 were no treatment trials. The order of trials was randomised between participants. Overall contingency between cue and outcome was zero. Table 2 shows the number of trials for each of the four possible trial types as a function of OD condition. On treatment trials, an image of a pill bottle was displayed at the center of the screen together with the text “Cloveritol”. Below the image, participants were shown “This patient was administered Cloveritol” in text. On no treatment trials, a greyed-out image of the same bottle is displayed with the text “No Treatment” and explicit instructions that the patient was administered no treatment. To maintain a similar key press response on each trial as in Experiments 1 & 2, participants had to press the space bar in order to proceed to the next screen, where they were asked to make a prediction about the patients’ chances of recovery. The instructions, prediction rating and subsequent outcome presentation were identical to that in Experiments 1 & 2.

**Table 2.** Total number of trials presented in Experiment 3 as a function of outcome density (OD) condition and trial type.

	Treatment		No Treatment	
	Patient recovered	Patient not recovered	Patient recovered	Patient not recovered
Low OD	5	20	3	12
High OD	20	5	12	3

### Method

#### Participants.

This study was approved by the University of New South Wales Human Research Ethics Advisory Panel C (Approval Number 3599). Four-hundred and six participants were recruited through the University of New South Wales (UNSW) undergraduate psychology pool (*M*_*age*_ = 19.9, *SD* = 3.66, Female = 267 female, Male = 129, Other = 4, NA / Choose not to say = 6). Participants completed the study online and received partial course credit for their participation. Allocation of participants to each of the six conditions was randomly determined by the experiment program.

#### Apparatus & Stimuli.

The apparatus and stimuli were the same as in Experiments 1 & 2.

#### Design & Procedure.

The design of Experiment 3 included three instruction conditions: a standard instruction group, a weak base-rate instruction group, and a strong base-rate instruction group, as well as two levels of outcome density (Low vs High OD). By including all three instructions in a single experiment, we were able to directly compare the effect of different degree of base-rate instructions on judgements about the efficacy of the treatment. The study design was fully between-subjects with six different conditions.

The procedure of Experiment 3 was very similar to Experiments 1 & 2 with the exception of participant cue administration. All participants were shown whether a patient was given Cloveritol or No Treatment at the start of each trial prior to making a prediction. All other procedural details were the same.

### Results

The only dependent variable in Experiment 3 was participants’ efficacy ratings at the end of the experiment. To determine if the type of instructions (standard, weak, strong base-rate instructions) influenced efficacy ratings, we ran planned contrasts comparing each of the base-rate instruction conditions to each other (weak vs strong instructions) as well as to the standard instruction condition individually (Bonferroni corrected alpha = .017). We also tested the effect of outcome density (Low vs High) and the interaction between OD and each of the instructions contrasts. Like in Experiments 1 & 2, we anticipate that any effect of instructions will be most evident in the High OD condition. Thus, we additionally tested the three instructions contrasts for Low and High OD conditions separately. Student *t* tests were again used to compare efficacy ratings to the veridical value of zero.

#### Exclusion Criteria.

**Figure 6.**
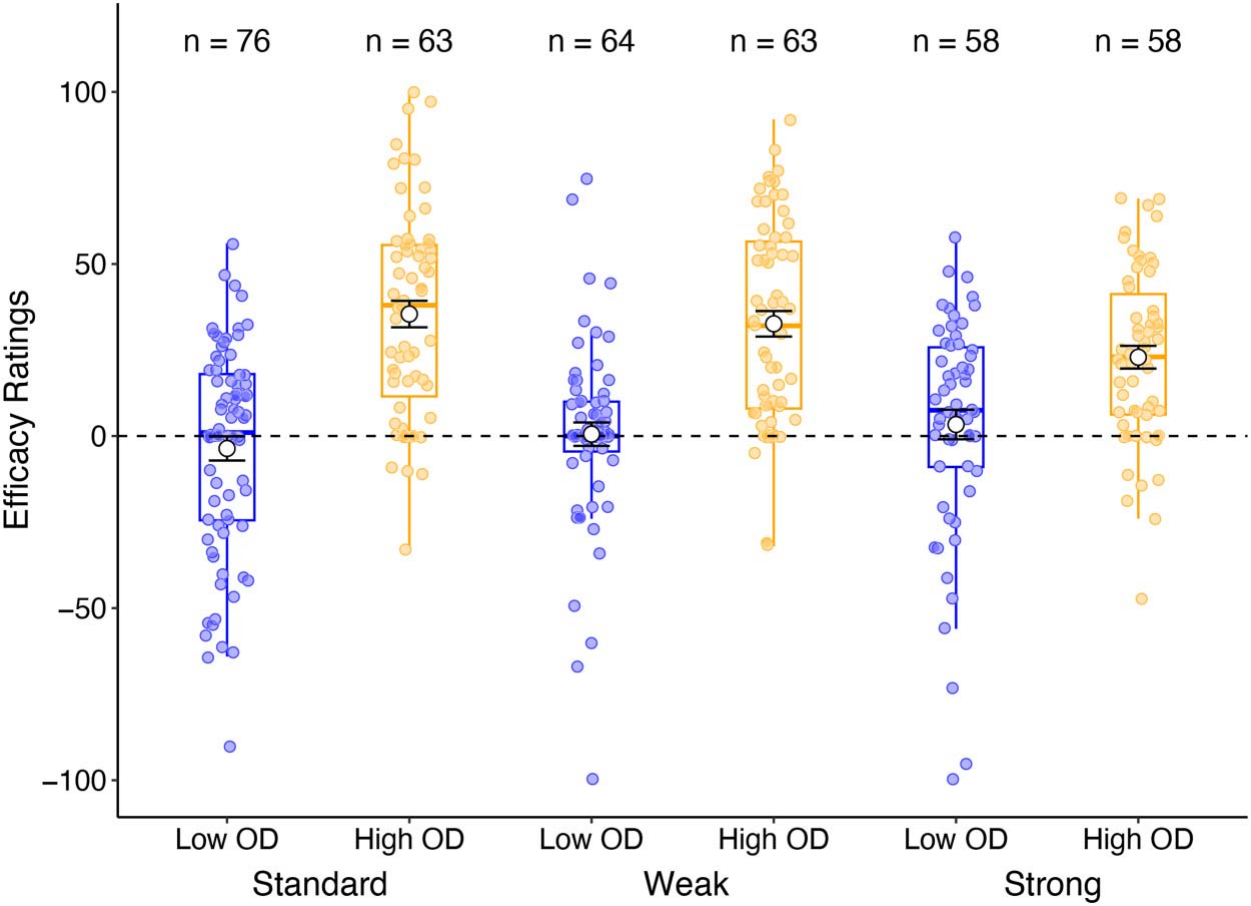
Average efficacy ratings provided at test (white circles illustrate the mean± standard error) as a function of outcome density (Low OD vs High OD) and base-rate instructions (Standard, Weak, Strong) for all participants in Experiment 3. Coloured circles indicate individual participant ratings.

We applied the same three exclusion criteria as in Experiment 2. After applying all exclusions, 382 datasets remained. Total number of participants in each condition after exclusions are shown at the top of Figure 6.

#### Efficacy Ratings.

Figure 6 illustrates participants’ efficacy ratings about the treatment as a function of instructions condition and OD. Consistent with Experiments 1 & 2, we again found a main effect of OD, *F*(1, 376) = 99.8, *p* < .001, *η*_p_^2^ = .210, BF_10_ = 42.4, with much greater illusory causation for participants in the High OD condition compared to the Low OD condition. Main effect contrasts revealed no difference in efficacy ratings for participants in the standard compared to the weak instruction condition, *F*(1, 376) = .034, *p* = .853, *η*_p_^2^ < .001, BF_10_ = −1.86, no significant difference between standard and strong instructions, *F*(1, 376) = .563, *p* = .453, *η*_p_^2^ = .001, BF_10_ = −1.96, and no significant difference between weak and strong instructions, *F*(1, 376) = .835, *p* = .361, *η*_p_^2^ = .002, BF_10_ = −1.65.

There was however an interaction between OD and the contrast comparing standard and strong instructions, *F*(1, 376) = 6.91, *p* = .009, *η*_p_^2^ = .018, BF_incl_ = 3.92. This interaction shows a much larger effect of OD in the standard instruction group compared to the strong instruction group, suggesting some benefit of the interactive base-rate instructions at reducing biases in learning due to the frequency of outcome-present trials. An interaction with OD was not found for the contrast comparing standard vs weak instructions, *F*(1, 376) = .932, *p* = .335, *η*_p_^2^ = .002, BF_incl_ = .283, and weak vs strong instructions, *F*(1, 376) = 2.74, *p* = .10, *η*_p_^2^ = .007, BF_incl_ = .735.

In order to determine if the interaction between OD and instructions was due to a reduction in efficacy ratings among High OD participants in the strong instruction group, we looked at main effect contrasts of instructions for the Low and High OD conditions separately, with our primary focus on the High OD condition. Here, we found only a marginal difference in ratings for standard vs strong instructions in the High OD condition, *F*(1, 181) = 5.81, *p* = .017, *η*_p_^2^ = .031, BF_10_ = 1.04. Thus, although there is some evidence that strong base-rate instructions might reduce the outcome density effect, evidence of this is inconclusive. All other analyses were not statistically significant, largest *F*(1, 181) = 3.48, *p* = .064, *η*_p_^2^ = .019, BF_10_ = .039. Student *t* test comparing average efficacy ratings against the veridical value of zero provided strong evidence of illusory causation among participants in the High OD condition across all three instructions conditions: standard, *t*(62) = 9.24, *p* < .001, *d* = 1.16, BF_10_ = 2.71e+10; weak base-rate instructions, *t*(62) = 8.77, *p* < .001, *d* = 1.11, BF_10_ = 4.64e+9; strong base-rate instructions, *t*(57) = 6.90, *p* < .001, *d* = .906, BF_10_ = 2.55e+6.

Experiment 3 showed no strong evidence that exposure to base-rate instructions in a passive contingency learning task was effective at directly reducing efficacy ratings for an ineffective treatment. There was an illusory causation effect in all three instruction conditions when participants were exposed to many trials where the patient recovered (High OD condition). This is further evidence that the frequency of the desired outcome occurring has a strong influence on causal judgements. We did however find a marginally significant reduction in causal ratings for participants in the strong instruction condition compared to standard instructions when outcome density was high. Together with the findings of Experiments 1 & 2, we conclude that simple reminders to consider the base-rate when evaluating causal relationships are not completely effective at reducing illusory belief in a contingency learning task where cue-outcome information is presented one at a time, suggesting that active reminders to engage in base-rate reasoning are not able to completely override persisting biases in memory when information is acquired incrementally. These studies show that the frequency of the outcome occurring has a strong influence on the strength of the causal illusion, and directly reducing the frequency in which participants choose to sample the cue may be the most effective means of reducing the bias, at least when information is encountered sequentially.

## EXPERIMENT 4: SINGLE-DECISION SAMPLING WITH SUMMARY INFORMATION

In Experiments 1–3, participants were presented with a trial-by-trial learning task where cue-outcome information was presented one at a time in a sequential fashion. This type of contingency learning is similar to how evidence is accumulated across many instances in our everyday lives. Another way of presenting information that is useful for making causal judgements is to present information in summary format. Summary information about the overall probability of the outcome with and without the cue present is useful as it allows the learner to accurately extract key information and quickly detect patterns in the data. Previous research has shown that outcome density effects still emerge under complete summary conditions where participants are simply given tabulated frequencies of the possible trial types (Kao & Wasserman, 1993). Double et al. (2020) also found a robust outcome density effect using a pseudo-summary format, where cue-outcome information was summarised into blocks such that each block consisted of three cue-present and three cue-absent trials, and each trial consisted of multiple outcomes associated with a single cue. Collectively, these studies suggest that the outcome density effect is robust and continues to influence causal judgements across a range of different presentation formats from trial-by-trial presentation of information to a complete summary, as well as hybrid formats that might better reflect what people encounter in some everyday contexts. However it is unclear whether strategies that aim to 1) reduce the sampling of cue-present trials, and 2) encourage base-rate comparison during causal reasoning would be present under summary presentation conditions.

In this study, we revert back to the active version of the contingency task, with the exception that participants were required to make a single decision about the number of patients they would administer the treatment to, with the remaining patients receiving no treatment. The total number of patients given the treatment or no treatment and subsequently recovered and not recovered were described on a single screen in text (summary format; see bottom panel of Figure 1 for example screenshots of the task). This summary presentation of information allowed us to determine whether effects of the instructional manipulation seen in Experiments 1 & 2 were consistent across different types of information-presentation formats. One possibility is that base-rate instructions are effective at helping people interpret simple summary information for causal judgements, however may not be adequate to correct persistent memory biases when information is accumulated over trials. Thus, we hypothesised that the benefit of base-rate instruction may be stronger under summary presentation format, indexed by significantly reduced treatment administration and weaker illusory causation amongst participants who received *some* base-rate information than those who received standard instructions. Like in Experiment 3, we included all three instructional manipulations and directly compared the effect of these instructions using planned contrasts.

### Methods

#### Participants.

Participants in Experiment 4 were recruited via Amazon’s Mechanical Turk, and participants were paid 3USD for the completion of the 10 min study. A total of 480 participants completed the study (*M*_*age*_ = 34.1, *SD* = 11.1, Female = 191, Male = 286, Other = 1, NA / Choose not to say = 2). Participant data were excluded based on the same exclusion criteria in previous experiments. Data were collected until there were close to 60 participants in each of the six groups. This led to a total of 386 usable datasets (158 females, *M*_age_ = 33.2, *SD* = 10.8); Low OD standard instructions *n* = 70, High OD standard instructions *n* = 69, Low OD weak base-rate instructions *n* = 61, High OD weak base-rate instructions *n* = 67, Low OD strong base-rate instructions *n* = 61, High OD strong base-rate instructions *n* = 58.

#### Design & Procedure.

Participants in this study were asked to make a single judgement on the number of patients (out of a possible total of 40 patients) to whom they would administer Cloveritol. They were told that the remaining patients would receive no treatment. This judgement was made on a linear scale from 0 to 40. Participants were then presented with information about patient outcomes in summary form: they were told the total number of patients administered Cloveritol who have recovered or did not recover, and the total number of patients given no treatment who either recovered or did not recover. Example screenshots are shown in the bottom panel of Figure 1. For participants in the Low OD condition, 20% of patients recovered from their illness and 80% of patients did not, and this was reversed for participants in the High OD condition. Importantly the probability of patient recovery was the same for patients administered Cloveritol and those given no treatment. Participants were then presented with a causal rating identical to that in the previous experiments.

### Results

A similar statistical analysis approach to previous experiments was used in this study on both efficacy ratings and the proportion of trials participants decided to administer the treatment. Importantly, the latter measure was presented as a single decision, in contrast to Experiments 1 & 2 where participants decided whether or not to administer the treatment on each trial. Like in previous experiments, we further evaluated the effect of instructions for Low and High OD conditions separately. We additionally compared the average number of patients treated in each instructional condition to the mean proportion of 0.5 to determine if the proportion of patients treated was significantly greater than half, and compared participants efficacy ratings to a value of zero.

#### Proportion of Treatment Administration.

**Figure 7.**
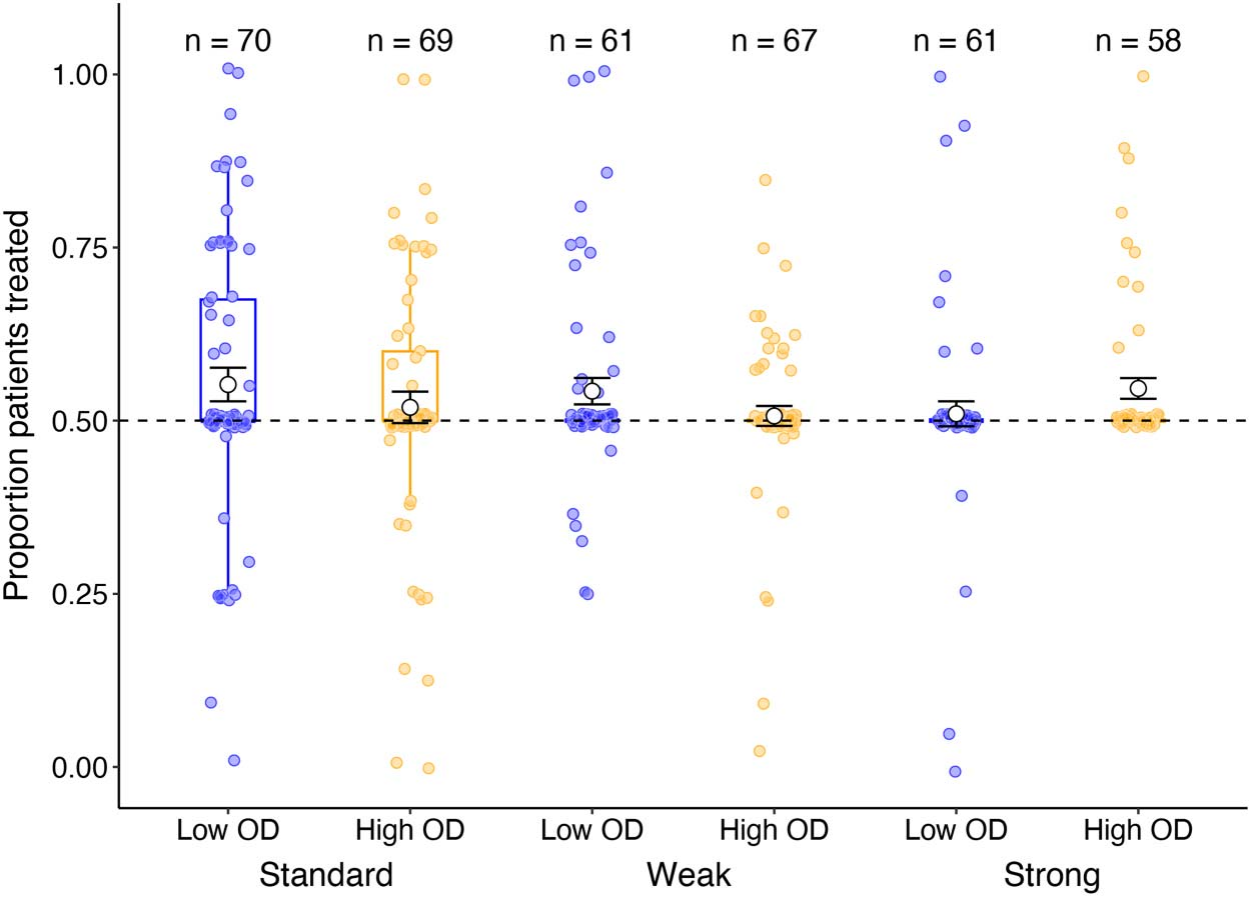
Proportion of patients treated with Cloveritol (± standard error) in a single choice task as a function of outcome density (OD) and instruction in Experiment 4.

The proportion of patients administered Cloveritol (of a total of 40 patients) as a function of OD and instruction condition are illustrated in Figure 7. Planned comparisons revealed no significant main effect contrasts, and no significant interactions between them, *largest F*(1, 380) = 3.26, *p* = .072, *η*_p_^2^ = .009, BF_incl_ = .323. Contrasts comparing the effects of base-rate instruction for each level of outcome density separately, also yielded no statistically significant findings, largest *F*(1, 380) = 2.34, *p* = .127, *η*_p_^2^ = .006, BF_10_ = .590.

Student *t*-tests comparing the average proportion of patients treated in each condition to a mean proportion of 0.5 revealed a significant difference in all conditions, however these differences were very small, with average proportions close to the 0.5 mark. The lack of difference between conditions could potentially be explained by a floor effect where the proportion of patients treated in the Standard condition was already close to 50% of the total number of patients. Thus there is little room for further reduction on this measure as a function of base-rate instruction. A summary of the statistical output from the *t*-test is presented in Table 3. Taken together, these results suggest that presentation of base-rate instructions did not significantly reduce cue administration when participants were asked to make a single decision on the total number of patients to be administered Cloveritol and no treatment.

**Table 3.** Summary statistics from Student *t*-test comparing the average proportion of patients treated to a mean proportion of 0.5 in Experiment 4.

	Mean	*SD*	*t*	df	*p*	Cohen’s d
Standard	.536	.196	2.15	138	.033	.182
Weak instructions	.524	.134	2.01	127	.047	.178
Strong instructions	.528	.130	2.33	118	.021	.214

#### Efficacy Ratings.

**Figure 8.**
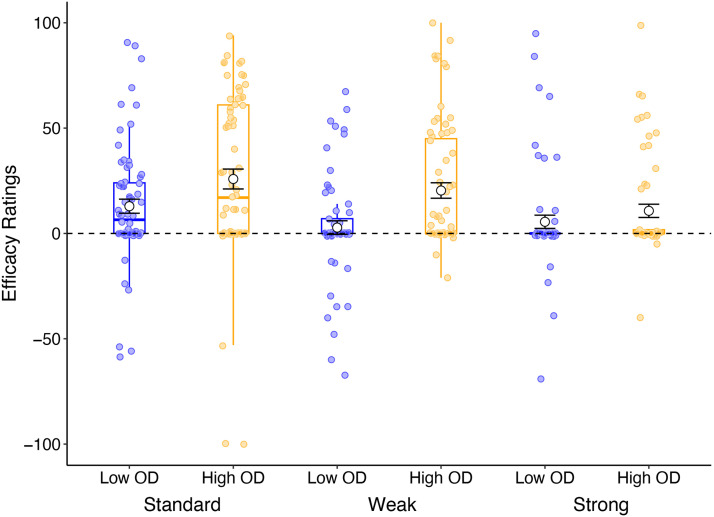
Average efficacy ratings (white circles illustrate the mean ± standard error) as a function of outcome density (OD) and instructions in Experiment 4.

Causal judgements about the efficacy of Cloveritol in treating the illness (relative to no treatment) is illustrated in Figure 8. Here, we found a main effect of OD, *F*(1, 380) = 15.9, *p* < .001, *η*_p_^2^ = .040, BF_10_ = 266.2, with significantly stronger illusions of causality in the High OD condition (*M* = 19.4, *SD* = 32.5) than the Low OD condition (*M* = 7.34, *SD* = 26.3). Planned contrasts comparing standard instructions to the strong instruction condition averaged over OD, showed a statistically significant effect of instructions, *F*(1, 380) = 9.45, *p* = .002, *η*_p_^2^ = .024, BF_10_ = 9.05, where evidence for the alternative hypothesis (difference in ratings between the two conditions) was 9 times more likely than the null. No other main effect or interactions were statistically significant, *largest F*(1, 380) = 4.73, *p* = .030, *η*_p_^2^ = .012, BF_10_ = .926 for the main effect contrast comparing efficacy ratings for participants in the weak instruction condition compared to standard instruction. Contrasts comparing the effects of instructions in the Low and High OD condition separately showed a significant difference in efficacy ratings when comparing standard to the strong instruction group in the High OD condition only, *F*(1, 380) = 8.38, *p* = .004, *η*_p_^2^ = .022, BF_10_ = 3.52. No other comparisons approached statistical significance, largest *F*(1, 380) = 3.91, *p* = .049, *η*_p_^2^ = .010, BF_10_ = 1.54 (contrast comparing standard to weak instructions for participants in the Low OD condition). Together, these results suggest that relative to no base-rate instruction, participants given strong base-rate instruction showed significantly weaker illusions of causality, and this effect was especially true for participants who saw a high overall proportion of patients recovering from the illness.

Student *t-tests* comparing participants’ efficacy ratings for High OD participants against a value of zero showed that average efficacy ratings were significantly above zero in all conditions: standard, *t*(68) = 5.47, *p* < .001, *d* = .658, BF_10_ = 21343, weak, *t*(66) = 5.56, *p* < .001, *d* = .680, BF_10_ = 28413, and strong instruction condition, *t*(57) = 3.41, *p* = .001, *d* = .448, BF_10_ = 23.3. As in the previous experiments, the inclusion of base-rate instructions did not completely eliminate illusory causation in the High OD condition. The same comparison in the Low OD condition however did reveal a statistically significant difference in the standard group, *t*(69) = 3.85, *p* < .001, *d* = .461, BF_10_ = 87.2, but not in either of the base-rate instruction conditions, *p* > .05, largest BF_10_ = .60. Thus there is a hint of a reduction in causal judgements for participants in the Low OD condition who were presented with *some* base-rate information compared to no base-rate information at all.

#### Mediation Analysis.

A mediation analysis of the relationship between the instruction manipulation and efficacy ratings was conducted with proportion of cue administration as the mediator and controlling for OD as a factor. Firstly, comparing the effect of *strong* base rate instructions to standard instructions on efficacy ratings, we found a significant total (direct + indirect) effect of strong base-rate instructions on efficacy ratings, *c* = −11.2, *p* = .003, 95% CI [−18.4, −3.96]. Here, efficacy ratings decrease as we move from standard to strong instructions. Importantly when we consider the direct and indirect effects separately, we found only a direct effect of instructions on efficacy ratings, *c*′ = −10.6, *p* = .002, 95% CI [−17.4, −3.86]. There was no significant effect of base-rate instructions on efficacy ratings via probability of cue administration, *a* * *b* = −0.52, *p* = .606, 95% CI [−3.23, 1.94].

Similarly, there was a significant total effect when comparing *weak* base-rate instructions to standard instructions, *c*_2_ = −7.67, *p* = .033, 95% CI [−14.7, −0.61], where we found evidence of a direct effect of instructions on efficacy ratings, *c*_2_ = −6.95, *p* = .041, 95% CI [−13.6, −0.29], but no indirect effect via probability of cue administration, *a*_2_ * *b*_2_ = −0.728, *p* = .550, 95% CI [−3.68, 1.74]. Overall, findings from Experiment 4 suggest that the presence of some form of base-rate instruction, relative to when no base-rate information was presented, was sufficient to reduce illusory causation when contingency information was presented in summary form, even when there was no influence of base-rate instruction on cue sampling. Unsurprisingly, the reduction in illusory belief appeared to be strongest when comparing participants in the strong base-rate instructions condition to the standard instructions condition, and when the frequency of the outcome occurring was also high. The benefit of the strong base-rate instructions could be due to the cumulative effect of presenting all three vignettes, or it could also potentially be the case that just a single vignette (for instance vignette #3, which is structurally most relevant to the causal illusion task we used here) would be as effective as providing all three; The three vignettes in the strong base-rate instructions differ in the nature of the information they provide and we cannot say with certainty what value each one provides. In addition, all participants were presented with all three vignettes and corrective feedback was provided in each case. Nevertheless, as in previous experiments, any reduction in illusory causation was partial, and the probability of the outcome occurring continued to exert a significant influence on causal beliefs.

## GENERAL DISCUSSION

Across four experiments, we tested whether providing purely text-based information highlighting the inherent value of base-rate comparison and its place in assessing medical evidence would reduce the illusion of causality. Unlike previous research on debiasing interventions that have provided extensive scientific training (e.g., Barberia et al., 2013; Blanco & Matute, 2019), our results showed some evidence that even simple instructions about the nature of clinical trials that only require a few minutes to read, were capable of modestly reducing illusory causal beliefs, particularly under high outcome density conditions where illusory causation was expected. We additionally show that these simple instructions were most effective at reducing illusory belief when information was presented in a summary format, which is one common way in which people receive information in the real world.

Presenting base-rate information could reduce biased causal judgments for a number of reasons. In this study, we have shown that the way in which base-rate instruction influences causal judgements might depend on the way in which the information is presented. When information was presented sequentially and participants could decide whether or not to administer the treatment to each patient (Experiments 1 & 2), we found the ability for base-rate instructions to reduce illusory beliefs was mediated by a reduction in the proportion of cue administration. We found no evidence of a direct effect of instructional manipulation on efficacy ratings, suggesting that under these conditions, base-rate instructions operate predominantly by encouraging normative cue-sampling behaviour (i.e., sampling equally between cue-present and cue-absent events), which increases their ability to detect a null contingency. These results were supported in Experiment 3, where participants had no choice over the activation of the cue. Under these conditions, we found no effect of base-rate instructions on causal belief ratings, with only a marginally significant reduction in efficacy ratings when comparing the strong instruction condition to the standard instruction group. Overall, these results showed that oversampling of cue information itself is correlated with heightened illusory beliefs. This finding is congruent with existing research on the effects of information sampling on causal judgements (Barberia et al., 2013).

In contrast, when information was presented in summary format in Experiment 4, there was a direct effect of strong base-rate instructions on reducing causal judgements relative to no base-rate instructions, despite normative cue-sampling in all groups. In other words, when participants had clear evidence that the probability of the outcome was not different for cue-present and cue-absent trials, there was still a tendency to rely on cue-present trials when making judgements about the efficacy of the treatment cue, unless they have been recently instructed on the use of base-rate comparison in causal judgements. These results suggest that instruction on the scientific method can attenuate causal illusions at two points in the cognitive process: firstly, as discussed above, it influences information seeking behaviour by reducing the bias to over-sample cue-present information, thereby increasing exposure to trials that conflict with the cue-outcome relationship (e.g., cue-absent/outcome-present events). Secondly, it influences how accumulated evidence is integrated and used to form causal judgements. Results of Experiment 4 suggests that even when participants were explicitly presented with accurate summary information about the cue-outcome relationship, they do not use the relevant information appropriately due to their bias to overweigh trials where both cue and outcome are present. The tendency to do this could be a product of participants assuming that cue-outcome coincidences are most important for inferring a causal relationship (Kao & Wasserman, 1993; Perales & Shanks, 2007; White, 2002), or because they are biased to base their beliefs only on confirmatory rather than disconfirmatory evidence (Wason, 1960). Whatever the mechanism underlying this bias in causal inference, our results suggest that reminders to engage in base-rate comparison are effective at attenuating this bias *during* contingency learning, and thus improving the veracity of causal judgements. This is in contrast to the mechanism thought to underlie the effectiveness of pre-training procedures on reducing causal illusions, where pre-training influences participants’ prior knowledge about the outcome base rate prior to exposure to the cue-outcome relationship (Blanco & Matute, 2019). Our finding has real-world import as it suggests that including simple base-rate reminders with summary information is an effective tool for encouraging scientific reasoning by helping people correctly interpret the information presented to them to form causal judgements.

Considering the results of all four experiments together, we propose that brief instructions on the scientific method for evaluating treatment efficacy may prove to be a useful tool in reducing illusory causal beliefs in two ways. Firstly, brief instructions are capable of mobilising behaviours that reduce oversampling of cue trials, such as preventing the overuse of the treatment without sufficient opportunity to compare its effects with a similar period of non-intervention. This has important implications for real-world health beliefs, where alternative therapies often do not produce any side effects (or any effect at all). Accordingly, people may be more liberal in using these complementary treatments since the short-term cost of treatment use is low (Blanco et al., 2014). People may thus develop strong beliefs about these ineffective treatments because they almost always use the treatment and have insufficient information about the probability of recovery when the treatment is not used. In such cases, even if the actual contingency between treatment and recovery is zero, the biased sampling of cue information (coupled with high overall base rate of recovery) might result in the individual experiencing contingency values that deviate from zero (Blanco et al., 2011). Secondly, results from Experiment 4 has shown that subtle reminders to engage in base-rate comparison may encourage individuals to increase their attention to cue-absent events when all available information is explicitly presented, thus more directly improving their ability to accurately detect a null-contingency between treatment use and health outcomes.

Finally, it is encouraging to observe that simple instructions about base-rates can reduce a pervasive bias that is widely observed in contingency learning. At the same time, it is perhaps concerning that the instructions had such a modest effect on efficacy ratings when the probability of the outcome occurring is high; we found significant illusory causation under High OD conditions when participants received standard instructions *and* when they received some instruction about the base-rate, despite the fact that the contingency learning phase came immediately after the instructions. Our findings are consistent with that of Barberia et al. (2013) and Blanco and Matute (2019), who similarly showed a persistent causal illusion after participants had undergone an extensive debiasing procedure. In the case of Barberia et al. (2013), the intervention involved an interactive lesson on the importance of a control condition in research design, and the role of base-rate comparison in inferring causal relationships. In Blanco and Matute (2019), a pre-training procedure was implemented prior to the contingency learning task, where participants first experienced the outcome occurring at a high base-rate without any cause present. On one hand, our results are promising since they suggest that simple and subtle reminders to engage in base-rate comparison are just as effective at reducing causal illusions compared to more extensive pre-training procedures. On the other hand, one can imagine that simple instructions of this nature would have an even more limited effect after a delay or for information garnered over a longer period of time. In fact, most real-world experiences with treatment and recovery involve accumulating evidence across many days, often with a single trial a day. There is some evidence to suggest that participants are capable of tracking the relationship between cues and outcomes across longer time periods but still fall for the illusion of causality when receiving one learning trial a day (Willett & Rottman, 2019). It is unclear what the effects of presenting information about base-rate comparisons are in these circumstances. Furthermore, the base-rate instructions presented in all experiments were related to evaluating the efficacy of a treatment, which was directly relevant to the experimental task. Although we do not expect the benefits of the instruction to be limited to this scenario, we are unable to conclude in this study that the scientific instruction provided would generalize to different causal relationships tested after a longer delay. Future research using tasks across different time frames and vignettes with different scenarios could be used to address the generalisability of these effects to other real-world illusory beliefs.

It is also worth noting that the present study involved instructing participants to evaluate a fictitious drug of which participants had no prior knowledge. Previous research has found an interactive effect of expectations and direct experience of cue-outcome events (Alloy & Tabachnik, 1984), with an asymmetrical contribution, such that people who had strong prior beliefs were less likely to change their causal judgements in light of contradictory covariational information (Fugelsang & Thompson, 2000), and were more likely to ‘explain away’ evidence that conflicts with their prior beliefs by invoking alternative unobserved causes (Chow et al., 2023; Luhmann & Ahn, 2007; Rottman et al., 2011). The influence of prior knowledge on causal beliefs is explicitly incorporated in Griffiths and Tenenbaum’s (2009) theory based causal induction, where causal inference is thought to be guided by both prior knowledge about the causal relationship, and an estimation of the strength of that causal relationship based on experienced information. In the context of the present study, despite Cloveritol being a fictitious treatment that participants are unfamiliar with, participants might hold domain-general knowledge about the effects of drugs on recovery, especially when they were instructed that the drug was being developed for that purpose. Thus, participants might come into the contingency learning task with positive expectations that the treatment is effective. In addition, under high outcome density zero contingency conditions, the covariation information provides little evidence for or against the causal relationship since there is little opportunity for the cue to increase the outcome above an already high baseline (Griffiths & Tenenbaum, 2005). This ambiguity in the experienced information may lead participants to rely on their existing positive prior causal belief when making causal judgements at the end of the study. This framework of thinking about causal induction provides one explanation for the findings in the present study, that is, base-rate instructions are insufficient to overcome participants’ strong prior beliefs about treatment efficacy and therefore fail to completely eliminate the causal illusion.

In the real world, individuals deciding whether to start a new treatment are likely to seek out information before commencing, and therefore likely acquire and hold some prior beliefs about the efficacy of the treatment when used for that purpose. Under a zero contingency, where the treatment is not efficacious, one would expect confirmation bias, or prior expectations, to play a larger role in biasing the detection of a meaningful relationship where none exists, especially when the outcome is potentially ambiguous, as is the case with fluctuating and often ambiguous health-related information (Blanco et al., 2020; Marsh & Ahn, 2009). It is also noteworthy that the two mechanisms we presented in preceding paragraphs on how base-rate instructions might influence causal judgements do not involve changing participants’ prior expectations, but rather they focus on how information is collected and integrated *during* contingency learning. It will be important for future research to address the influence of prior expectations on causal reasoning to determine if strategies implemented to reduce illusory beliefs are effective in doing so when the learner has weak vs strong prior expectations and motivation to detect a meaningful causal relationship between treatment use and recovery.

In summary, we found that presenting brief instructions on using base-rate information when inferring a causal relationship is sufficient to modestly reduce illusory causation for an ineffective treatment. This effect was particularly evident when the overall base-rate of the outcome was high, a condition that reliably inflates illusory belief. Instruction on the scientific method attenuated illusory causation in two ways: firstly, base-rate instructions encouraged people to adopt more appropriate information sampling strategies, and secondly by directly scaffolding scientific reasoning processes by reducing the bias to overweigh or only attend to cue-present information. Our findings highlight the benefit of including subtle reminders on the value of base-rate information immediately prior to evaluating treatment efficacy for more effective decision-making. This is highly relevant in the modern information age where individuals are inundated with a wealth of information about various treatments and their proposed health effects. Thus, the ability to critically evaluate the validity of the claims made about these treatments may be a useful tool in improving decision-making. Causal illusions in medical decision-making may result in detrimental effects not only to the individual patient who chooses to use an ineffective treatment in place of a scientifically-validated one (Freckelton, 2012), but may also influence public healthcare systems (Wiese et al., 2010). These findings have important implications for encouraging analytical thinking in the evaluation of medical treatments, particularly in the case of pseudo-medicine where treatments are not efficacious but the conditions under which these treatments are used readily lend themselves to illusory beliefs.

## FUNDING INFORMATION

This research was supported by Discovery Grant DP150104267 and DP190100410 from the Australian Research Council.

## AUTHOR CONTRIBUTIONS

J.C. contributed to study design, data collection, analyses and manuscript preparation. M.G. and E.L. contributed to study design and manuscript preparation. B.C. contributed to manuscript preparation. All authors read and approved the final manuscript.

## OPEN PRACTICES STATEMENT

The datasets generated during and analysed in the current study are available at Open Science Framework https://osf.io/9wckv/, and none of the experiments was preregistered.

## Supplementary Material



## References

[bib1] Allan, L. G. (1980). A note on measurement of contingency between two binary variables in judgment tasks. Bulletin of the Psychonomic Society, 15(3), 147–149. 10.3758/BF03334492

[bib2] Alloy, L. B., & Abramson, L. Y. (1979). Judgment of contingency in depressed and nondepressed students: Sadder but wiser? Journal of Experimental Psychology: General, 108(4), 441–485. 10.1037/0096-3445.108.4.441, 528910

[bib3] Alloy, L. B., & Tabachnik, N. (1984). Assessment of covariation by humans and animals: The joint influence of prior expectations and current situational information. Psychological Review, 91(1), 112–149. 10.1037/0033-295X.91.1.112, 6571422

[bib11] Barberia, I., Blanco, F., Cubillas, C. P., & Matute, H. (2013). Implementation and assessment of an intervention to debias adolescents against causal illusions. PLoS One, 8(8), e71303. 10.1371/journal.pone.0071303, 23967189 PMC3743900

[bib4] Barrett, B., Brown, R., Rakel, D., Mundt, M., Bone, K., Barlow, S., & Ewers, T. (2010). Echinacea for treating the common cold: A randomized trial. Annals of Internal Medicine, 153(12), 769–777. 10.7326/0003-4819-153-12-201012210-00003, 21173411 PMC3056276

[bib5] Blanco, F., Barberia, I., & Matute, H. (2014). The lack of side effects of an ineffective treatment facilitates the development of a belief in its effectiveness. PLoS One, 9(1), e84084. 10.1371/journal.pone.0084084, 24416194 PMC3885525

[bib7] Blanco, F., & Matute, H. (2015). Exploring the factors that encourage the illusions of control: The case of preventive illusions. Experimental Psychology, 62(2), 131–142. 10.1027/1618-3169/a000280, 25384640 PMC4614377

[bib6] Blanco, F., & Matute, H. (2019). Base-rate expectations modulate the causal illusion. PLoS One, 14(3), e0212615. 10.1371/journal.pone.0212615, 30835775 PMC6400408

[bib9] Blanco, F., Matute, H., & Vadillo, M. A. (2011). Making the uncontrollable seem controllable: The role of action in the illusion of control. Quarterly Journal of Experimental Psychology, 64(7), 1290–1304. 10.1080/17470218.2011.552727, 21432736

[bib8] Blanco, F., Matute, H., & Vadillo, M. A. (2013). Interactive effects of the probability of the cue and the probability of the outcome on the overestimation of null contingency. Learning & Behavior, 41(4), 333–340. 10.3758/s13420-013-0108-8, 23529636

[bib10] Blanco, F., Moreno-Fernández, M. M., & Matute, H. (2020). Are the symptoms really remitting? How the subjective interpretation of outcomes can produce an illusion of causality. Judgment and Decision Making, 15(4), 572–585. 10.1017/S1930297500007506

[bib12] Chow, J. Y. L., Colagiuri, B., & Livesey, E. J. (2019). Bridging the divide between causal illusions in the laboratory and the real world: The effects of outcome density with a variable continuous outcome. Cognitive Research: Principles & Implications, 4(1), 1–15. 10.1186/s41235-018-0149-9, 30693393 PMC6352562

[bib13] Chow, J. Y. L., Colagiuri, B., Rottman, B. M., Goldwater, M., & Livesey, E. J. (2021). Pseudoscientific health beliefs and the perceived frequency of causal relationships. International Journal of Environmental Research and Public Health, 18(21), 11196. 10.3390/ijerph182111196, 34769714 PMC8583395

[bib14] Chow, J. Y. L., Lee, J. C., & Lovibond, P. F. (2023). Using unobserved causes to explain unexpected outcomes: The effect of existing causal knowledge on protection from extinction by a hidden cause. Journal of Experimental Psychology: Learning, Memory, and Cognition. 10.1037/xlm0001306, 38095948

[bib15] de Leeuw, J. R. (2015). jsPsych: A Javascript library for creating behavioral experiments in a Web browser. Behavior Research Methods, 47(1), 1–12. 10.3758/s13428-014-0458-y, 24683129

[bib16] Double, K. S., Chow, J. Y. L., Livesey, E. J., & Hopfenbeck, T. N. (2020). Causal illusions in the classroom: How the distribution of student outcomes can promote false instructional beliefs. Cognitive Research: Principles and Implications, 5(1), 34. 10.1186/s41235-020-00237-2, 32748083 PMC7399015

[bib17] Fugelsang, J. A., & Thompson, V. A. (2000). Strategy selection in causal reasoning: When beliefs and covariation collide. Canadian Journal of Experimental Psychology/Revue Canadienne de Psychologie Expérimentale, 54(1), 15–32. 10.1037/h0087327, 10721236

[bib18] Freckelton, I. (2012). Death by homeopathy: Issues for civil, criminal and coronial law and for health service policy. Journal of Law and Medicine,19(3), 454–478. 22558899

[bib19] Griffiths, T. L., & Tenenbaum, J. B. (2005). Structure and strength in causal induction. Cognitive Psychology, 51(4), 334–384. 10.1016/j.cogpsych.2005.05.004, 16168981

[bib20] Griffiths, T. L., & Tenenbaum, J. B. (2009). Theory-based causal induction. Psychological Review, 116(4), 661–716. 10.1037/a0017201, 19839681

[bib21] Hayes, A. F. (2017). Introduction to mediation, moderation, and conditional process analysis: A regression-based approach. Guilford publications.

[bib22] Kao, S.-F., & Wasserman, E. A. (1993). Assessment of an information integration account of contingency judgment with examination of subjective cell importance and method of information presentation. Journal of Experimental Psychology: Learning, Memory, and Cognition, 19(6), 1363–1386. 10.1037/0278-7393.19.6.1363

[bib23] Karsch-Völk, M., Barrett, B., Kiefer, D., Bauer, R., Ardjomand-Woelkart, K., & Linde, K. (2014). Echinacea for preventing and treating the common cold. Cochrane Database of Systematic Reviews, 2014(2), CD000530. 10.1002/14651858.CD000530.pub3, 24554461 PMC4068831

[bib24] Le Pelley, M. E., Griffiths, O., & Beesley, T. (2017). Associative accounts of causal cognition. In M. R. Waldmann (Ed.), The Oxford handbook of causal reasoning (pp. 13–28). Oxford University Press. 10.1093/oxfordhb/9780199399550.013.2

[bib25] Lilienfeld, S. O., Ritschel, L. A., Lynn, S. J., Cautin, R. L., & Latzman, R. D. (2014). Why ineffective psychotherapies appear to work: A taxonomy of causes of spurious therapeutic effectiveness. Perspectives on Psychological Science, 9(4), 355–387. 10.1177/1745691614535216, 26173271

[bib26] Lim, A., Cranswick, N., & South, M. (2011). Adverse events associated with the use of complementary and alternative medicine in children. Archives of Disease in Childhood, 96(3), 297–300. 10.1136/adc.2010.183152, 21178176

[bib27] Luhmann, C. C., & Ahn, W.-K. (2007). BUCKLE: A model of unobserved cause learning. Psychological Review, 114(3), 657–677. 10.1037/0033-295X.114.3.657, 17638500 PMC2659393

[bib28] Mandel, D. R., & Lehman, D. R. (1998). Integration of contingency information in judgments of cause, covariation, and probability. Journal of Experimental Psychology: General, 127(3), 269–285. 10.1037/0096-3445.127.3.269

[bib29] Marsh, J. K., & Ahn, W.-K. (2009). Spontaneous assimilation of continuous values and temporal information in causal induction. Journal of Experimental Psychology: Learning, Memory, and Cognition, 35(2), 334–352. 10.1037/a0014929, 19271850 PMC2826811

[bib30] Matute, H., Yarritu, I., & Vadillo, M. A. (2011). Illusions of causality at the heart of pseudoscience. British Journal of Psychology, 102(3), 392–405. 10.1348/000712610X532210, 21751996

[bib31] Morey, R., & Rouder, J. (2022). BayesFactor: Computation of Bayes factors for common designs. R package version 0.9.12-4.4. https://CRAN.R-project.org/package=BayesFactor

[bib32] Perales, J. C., & Shanks, D. R. (2007). Models of covariation-based causal judgment: A review and synthesis. Psychonomic Bulletin & Review, 14(4), 577–596. 10.3758/BF03196807, 17972719

[bib33] Rouder, J. N., Morey, R. D., Speckman, P. L., & Province, J. M. (2012). Default Bayes factors for ANOVA designs. Journal of Mathematical Psychology, 56(5), 356–374. 10.1016/j.jmp.2012.08.001

[bib34] Rouder, J. N., Morey, R. D., Verhagen, J., Swagman, A. R., & Wagenmakers, E.-J. (2017). Bayesian analysis of factorial designs. Psychological Methods, 22(2), 304–321. 10.1037/met0000057, 27280448

[bib35] Rottman, B. M., Ahn, W. K., & Luhmann, C. C. (2011). When and how do people reason about unobserved causes. In P. M. Illari, F. Russo, & J. Williamson (Eds.), Causality in the sciences (pp. 150–183). Oxford University Press. 10.1093/acprof:oso/9780199574131.003.0008

[bib36] Shanks, D. R., & Dickinson, A. (1988). Associative accounts of causality judgment. In G. H. Bower (Ed.), Psychology of learning and motivation (Vol. 21, pp. 229–261). Academic Press. 10.1016/S0079-7421(08)60030-4

[bib37] Steel, A., McIntyre, E., Harnett, J., Foley, H., Adams, J., Sibbritt, D., Wardle, J., & Frawley, J. (2018). Complementary medicine use in the Australian population: Results of a nationally-representative cross-sectional survey. Scientific Reports, 8(1), 17325. 10.1038/s41598-018-35508-y, 30470778 PMC6251890

[bib38] Thorwart, A., & Livesey, E. J. (2016). Three ways that non-associative knowledge may affect associative learning processes. Frontiers in Psychology, 7, 2024. 10.3389/fpsyg.2016.02024, 28082943 PMC5186804

[bib39] Wason, P. C. (1960). On the failure to eliminate hypotheses in a conceptual task. Quarterly Journal of Experimental Psychology, 12(3), 129–140. 10.1080/17470216008416717

[bib40] White, P. A. (2002). Perceiving a strong causal relation in a weak contingency: Further investigation of the evidential evaluation model of causal judgement. Quarterly Journal of Experimental Psychology Section A, 55(1), 97–114. 10.1080/02724980143000181, 11873858

[bib41] Wiese, M., Oster, C., & Pincombe, J. (2010). Understanding the emerging relationship between complementary medicine and mainstream health care: A review of the literature. Health, 14(3), 326–342. 10.1177/1363459309358594, 20427637

[bib42] Willett, C., & Rottman, B. M. (2019). The accuracy of causal learning over 24 days. In A. Goel, C. Seifert, & C. Freska (Eds.), Proceedings of the 41st annual conference of the cognitive science society. Cognitive Science Society.

