# Supplementary material for: Instruction on the Scientific Method Provides (Some) Protection Against Illusions of Causality
Source: Open Mind (Camb). 2024 May 10;8:639–65. doi: 10.1162/opmi_a_00141 (PMC11142631; doi:10.1162/opmi_a_00141)
Supplement: Supplementary file 1 [file opmi-08-639-s001.docx]

Supplementary Materials

**Table S1**

*Number of excluded datasets by exclusion criteria in each of the four reported experiments.*

|  | Instruction repeats > 2 | Admitted to writing down information | Selected wrong option  Bot check (Exp1) /  Memory check (Exp 2-4) |
| --- | --- | --- | --- |
| Experiment 1 (N = 279) | | | |
|  | 9 | 24 | 1 |
| Experiment 2 (N = 290) | | | |
|  | 24 | 17 | 1 |
| Experiment 3 (N = 406) | | | |
|  | 19 | 6 | 2 |
| Experiment 4 (N = 480) | | | |
|  | 56 | 78 | 20 |

*Note. There may be overlapping datasets across the three exclusion conditions.*

**Experiments 1 & 2**

**Table S2**

*Average experienced Δp as a function of instructional condition and OD condition, separated by Experiment. There was no statistically significant difference in average Δp as a function of group.*

|  |  | Standard Instructions | | Base-Rate Instructions | |
| --- | --- | --- | --- | --- | --- |
|  |  | Mean | SD | Mean | SD |
| Experiment 1 | Low OD | .016 | .131 | -.003 | .125 |
|  | High OD | -0.01 | .185 | .004 | .174 |
| Experiment 2 | Low OD | .013 | .118 | -.027 | .137 |
|  | High OD | .007 | .238 | .015 | .170 |

Analysis S1

***Prediction ratings across training***

Across the training phase, participants were required to first decide whether to administer the treatment or no treatment to the patient. Following this, they were instructed to make a prediction on the likelihood that the patient will recover given their choice, on a sliding scale from ‘Very Unlikely’ to ‘Very Likely’. This scale was converted to a numerical scale from 0-100 for analysis. We calculated participants’ average prediction ratings for treatment trials and no treatment trials and analysed these as a function of OD and instructions. Participants who failed to select either the treatment cue or no treatment at least once in the 40 trials were removed from analysis.

**Figure S1.**

*Average Outcome Predictions (+/-SEM) as a Function of Cue Type, Instructions and Outcome Density Condition in Experiments 1 and 2.*


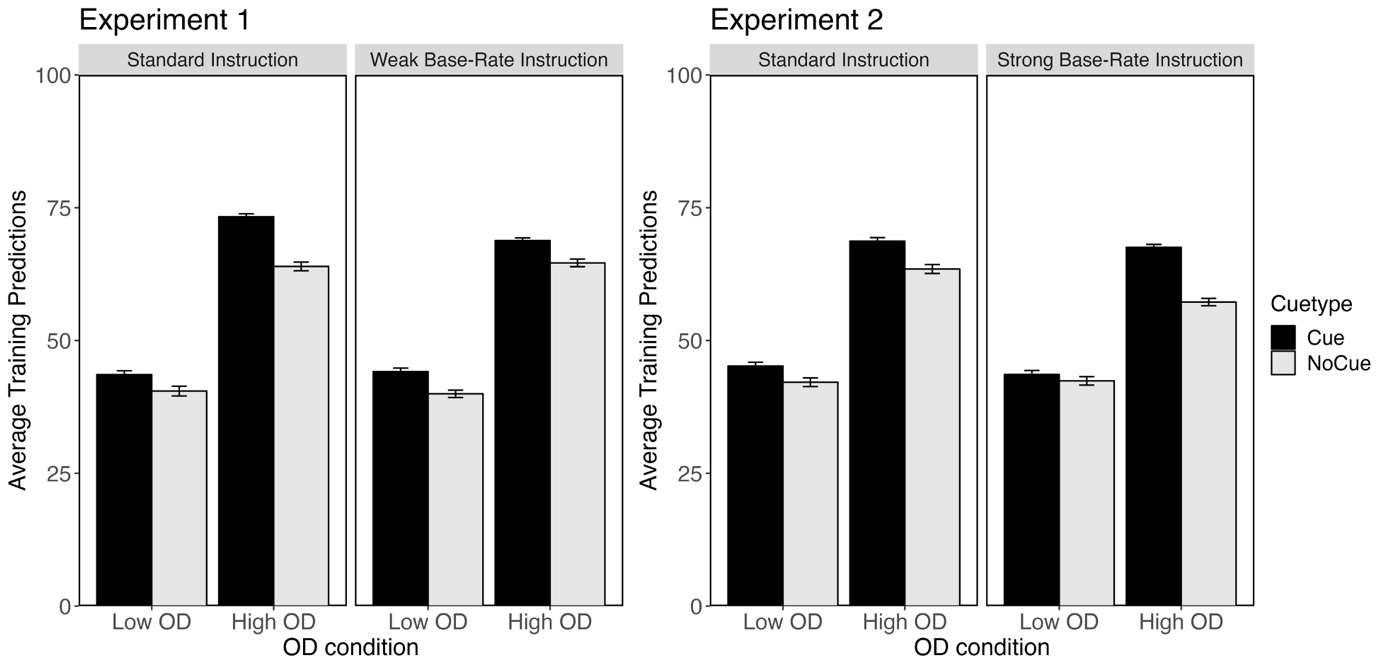


***Experiment 1.*** Average prediction ratings during training in Experiment 1 is shown on the left panel of Figure S1. For completeness, we have also included an illustration of the average ratings on each trial as a function of cue, OD and instructions in Experiment 1 (see Figure S2).

A 2 (OD) x 2 (instruction) x (2) (Cue Type) mixed-model analysis of variance revealed a significant main effect of cue type, *F*(1,221) = 41.7, *p* < .001, η_p_^2^ = .159, BF_10_ = 3.28e+6, and a significant interaction between cue type and OD, *F*(1,221) = 4.95, *p* = .027, η_p_^2^ = .022, BF_incl_ = 1.19. There was also a main effect of OD, *F*(1,221) = 193.1, *p* < .001, η_p_^2^ = .466, BF_10_ = 6.36e+28, with significantly higher prediction ratings for participants in the High OD condition (M = 65.9, SD = 13.86) compared to the Low OD condition (M = 41.7, SD = 16.7). Together these results suggest a bias towards providing higher prediction ratings on cue trials (M = 56.7, SD = 19.5) compared to no cue trials (M = 49.7, SD = 19.9), and this difference was more pronounced when the frequency of the outcome occurring was high than when it was low. There was no main effect or interaction involving the instruction

manipulation, largest *F*(1,221) = 2.86, *p* = .092, η_p_^2^ = .013, BF_10_ = .451.

***Experiment 2.*** Average predictions during training in Experiment 2 are shown on the right panel of Figure S1. Average predictions on each trial is shown in Figure S3.

In Experiment 2, we again found a significant main effect of cue type, *F*(1,236) = 21.6, *p* < .001, η_p_^2^ = .084, BF_10_ = 762.3, and a significant interaction between cue type and OD, *F*(1,236) = 8.44, *p* = .004, η_p_^2^ = .035, BF_incl_ = 7.26. There was again a main effect of OD, *F*(1,236) = 117.4, *p* < .001, η_p_^2^ = .332, BF_10_ = 2.50e+19. Like in Experiment 1, we found no main effect or interaction involving the instruction manipulation, largest *F*(1,236) = 1.78, *p* = .183, η_p_^2^ = .007, BF_incl_ = .419. Results from both experiments showed a reliable difference in predictions towards Cloveritol and No Treatment trials, and an effect of the outcome density manipulation. However in both experiments we did not find any effect of the instructional manipulation of participants predictions.

**Figure S2.**

*Average Outcome Predictions (+/-SEM) as a Function of Cue Type Selected on that Trial, Instructions and Outcome Density Condition in Experiment 1.*

**
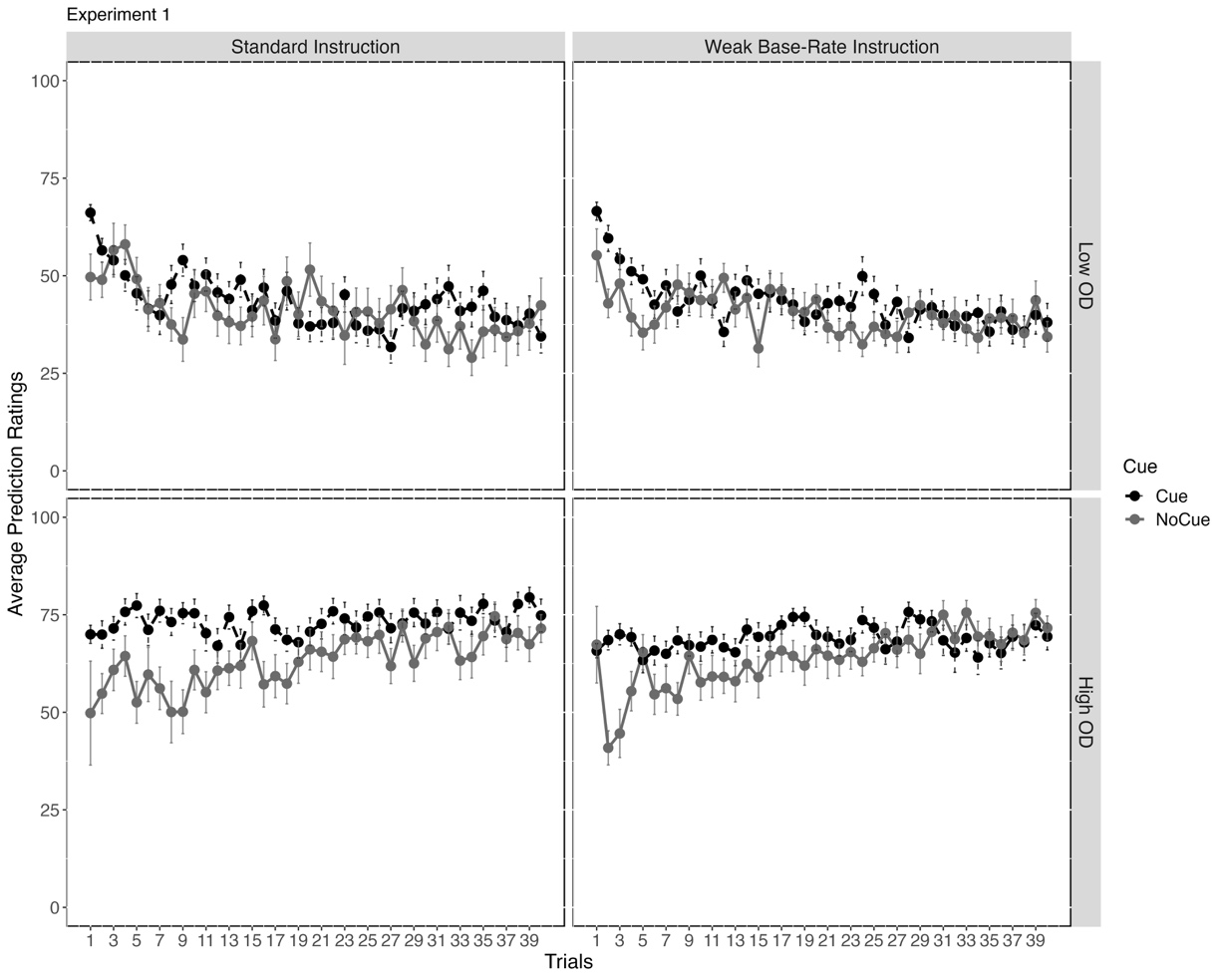
**

*Note.* Augmented instructions in Experiment 1 was a single page reminder about the logic of base-rate comparison in a randomised controlled trial.

**Figure S3**

*Average Outcome Predictions (+/-SEM) as a Function of Cue Type Selected on that Trial, Instructions and Outcome Density Condition in Experiment 2.*


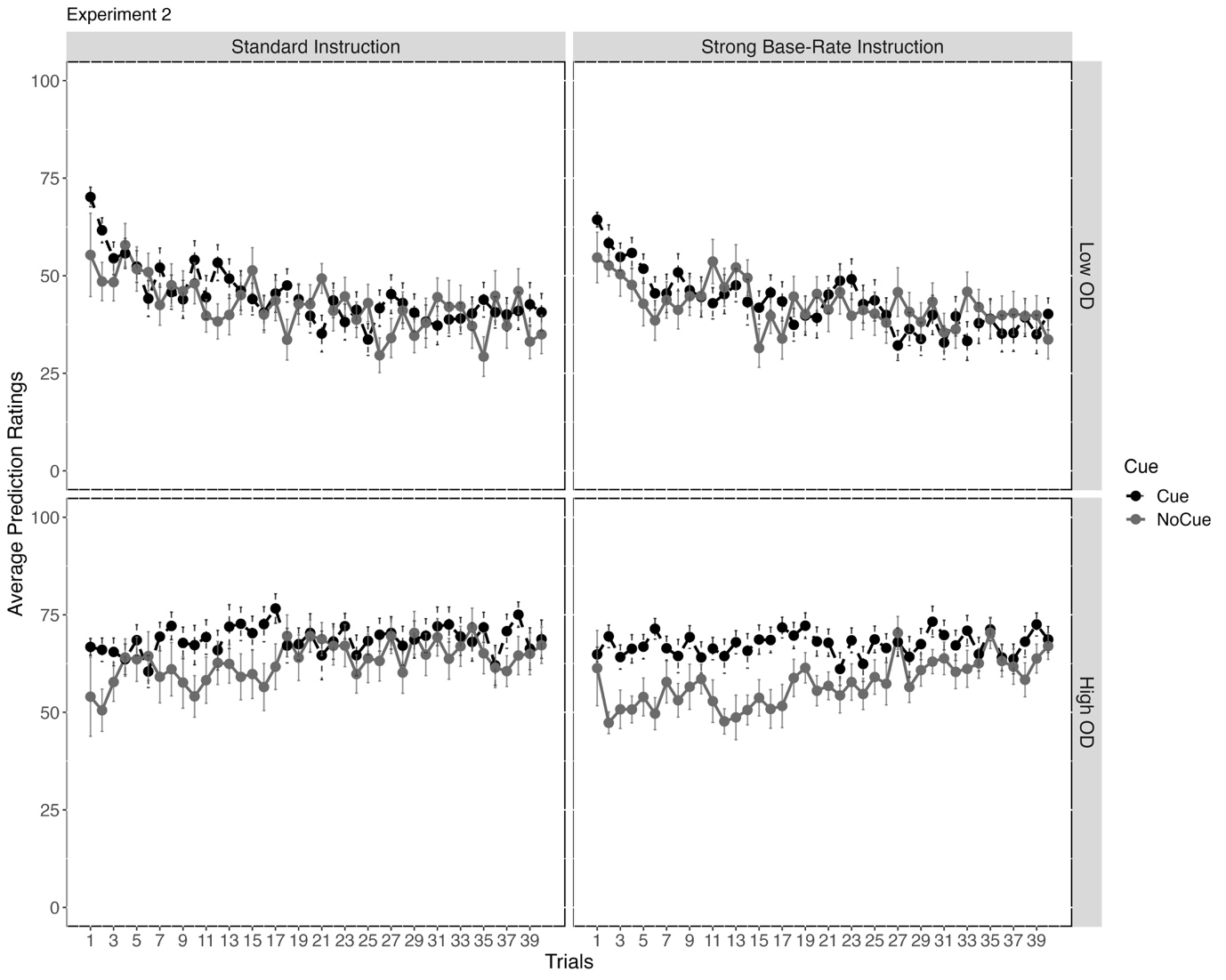


*Note.* Augmented instructions in Experiment 2 included practice vignettes evaluating the efficacy of a fictitious treatment (with feedback provided).

**Experiment 3**

***Predictions across training***

All participants in Experiment 3 experienced 25 Cue trials and 15 No Cue trials intermixed across training. Average predictions for Cue and No Cue trials separated by OD and instruction conditions are shown in Figure S4, and average predictions by trial is depicted in Figure S5.

Analysis of average prediction as a function of Cue type (2), OD and instruction contrast comparing standard to weak prime and standard to strong prime instructions found only a main effect of Cue type, F(1,376) = 148.8, *p* < .001, η_p_^2^ = .284, BF_10_ = 1.39e+19, a main effect of OD, F(1,376) = 492, *p* < .001, η_p_^2^ = .567, BF_10_ = 5.62e+98, and an interaction between the two factors, F(1,376) = 12.9, *p* < .001, η_p_^2^ = .033, BF_incl_ = 9.45. We found no significant difference in prediction ratings as a function of instruction condition, nor were there any interactions with instruction as a factor, *largest F*(1,376) = 2.16, *p* = .143, η_p_^2^ = .006, BF_incl_ = .299. Although no effects of instruction were found on trial-by-trial predictions, this is not entirely inconsistent with the findings from participants’ efficacy ratings, where we found only anecdotal evidence of a direct effect of base-rate instructions on causal beliefs under these conditions. In addition, previous studies have reported differences in the types of information participants used to form momentary predictions compared to when forming a causal judgement about all the information presented (Vadillo et al., 2005). Thus, it is plausible that the intervention implemented in this study has stronger impact on participants’ information-seeking behaviour, as shown in Experiments 1 & 2, and only very marginal impact on their final causal judgements, and no effect at all on their predictions across trials.

**Figure S4**

*Average Outcome Predictions (+/-SEM) as a Function of Cue Type, Instructions and Outcome Density Condition in Experiment 3.*


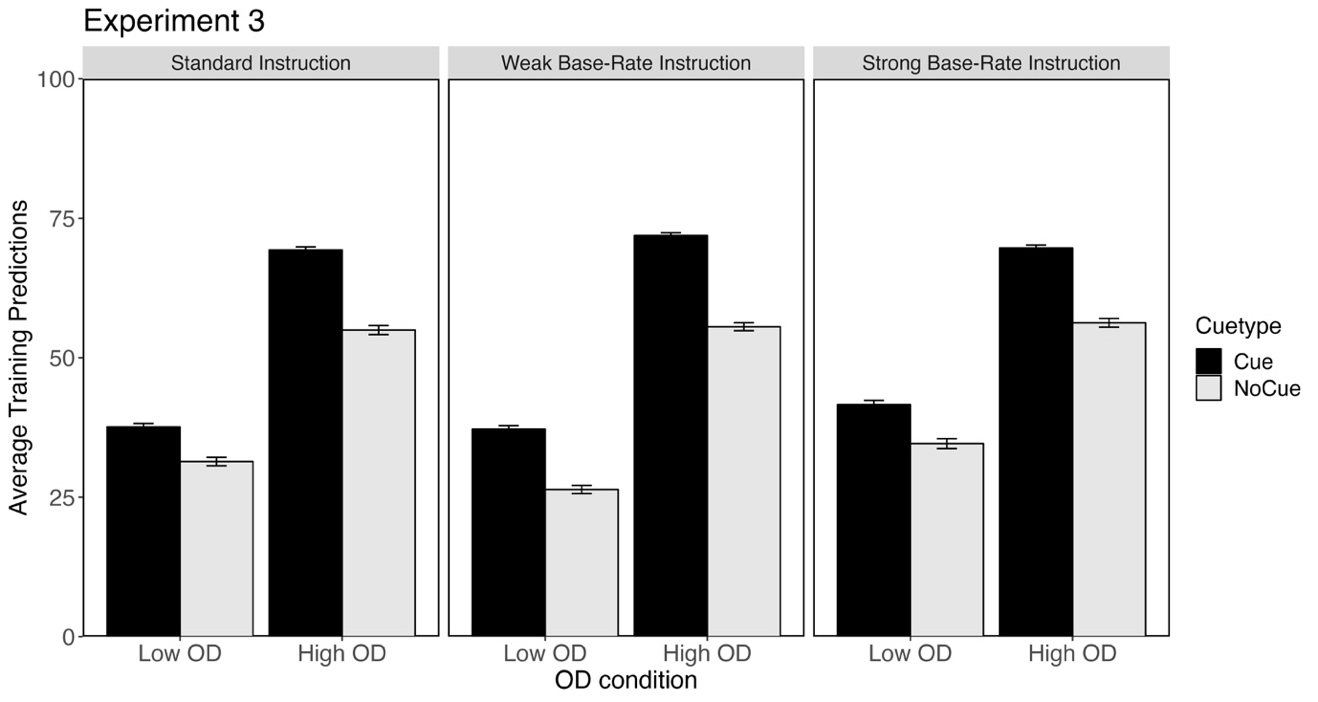


**Figure S5**

*Average Outcome Predictions (+/-SEM) as a Function of Cue Type, Trial, Instructions and Outcome Density Condition in Experiment 3. Presentation of cue and no cue trials were intermixed across training, with a total of 25 cue presentations and 15 no cue presentations.*


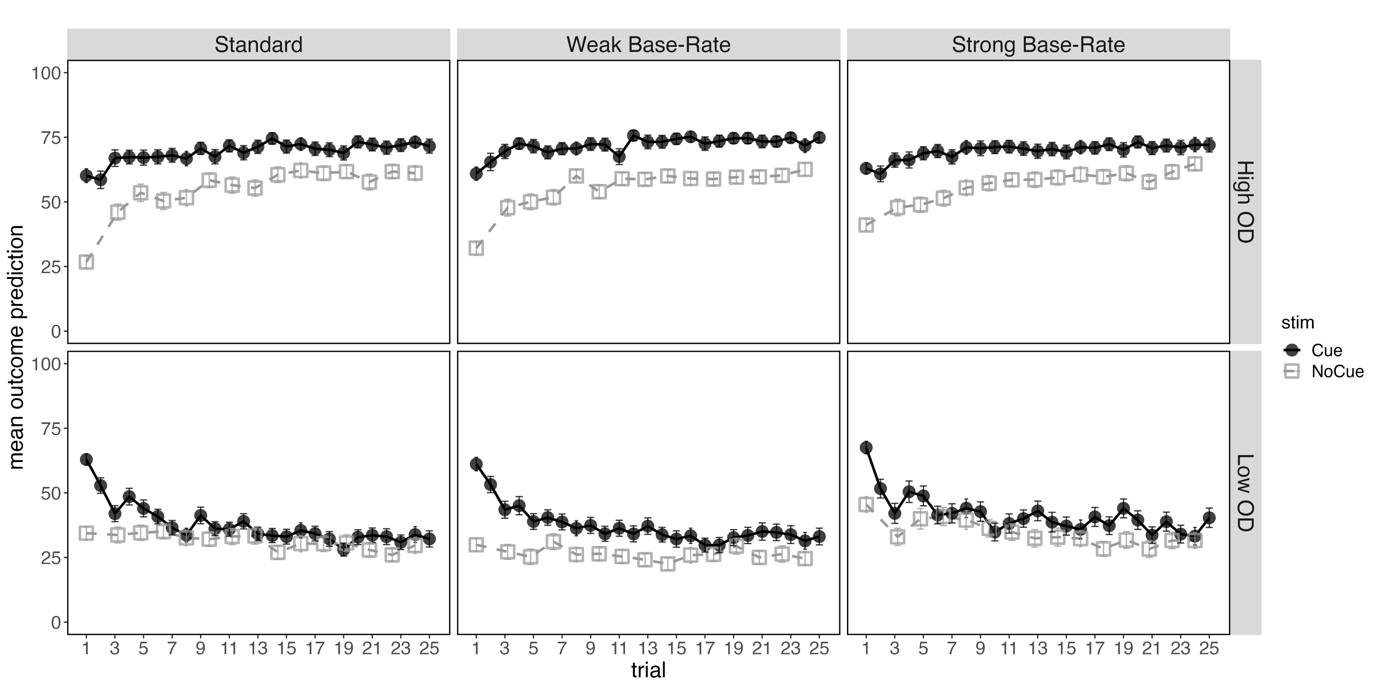


Materials S1

**Vignettes used in strong base-rate instruction conditions in Experiments 2, 3 & 4**

**Vignette 1:**

Recently, there was a lot of excitement about a research study suggesting that new formulation of B12 vitamins, B12tac, could help people who suffer from migraine headaches. Here is a description of the research:

“200 people who suffer from migraine headaches were given B12tac for one week. 87% of the people reported their symptoms were reduced by the end of the week, and that they then felt more productive at work, and more relaxed at home. The researchers concluded that B12tac is an effective drug to treat migraine headaches.”

Do the researchers have good evidence for the conclusion that B12tac effectively treats migraines?

o Yes

o No

Feedback:

**Even though 87% recovery rate sounds very high, the researchers do not know what percentage of people would have recovered without the drug. Therefore we might conclude NO, they did not have good evidence.**

**Vignette 2:**

A competing drug company was also developing a treatment based on B vitamins, called BetaTX for migraine headaches. Their research is described below:

“200 people who suffer from migraine headaches were given BetaTX for one week, and an

additional 200 people who suffer from migraine headaches were not given the experimental

drug. 79% of the people who took the drug reported their symptoms were reduced by the end

of week, while only 45% of the people who did not take the drug reported their symptoms were

reduced by the end of the week. The researchers concluded that BetaTX is an effective drug to

treat migraine headaches.”

Do the researchers have good evidence for the conclusion that BetaTX effectively treats migraines?

o Yes

o No

Feedback:

**A higher rate of recovery was observed in those people who took the drug than those who did not. Therefore we might conclude YES, they have good evidence.**

**Vignette 3:**

Based on the earlier success of BetaTX for migraine headaches, the same drug company wanted to see if it would also reduce chronic back pain.

“200 people who suffer from chronic back pain were given BetaTX for one week, and an

additional 200 people who suffer from chronic back pain were not given the experimental drug. 64% of the people who took the drug reported their symptoms were reduced by the end of the week, and 66% of the people who did not take the drug reported their symptoms were reduced by the end of the week.”

Do the researchers have good evidence for the conclusion that BetaTX effectively treats chronic back pain?

o Yes

o No

Feedback:

**Although the majority of people who took the drug improved, the same was true for the people who did not take the drug. Therefore we might conclude NO, they did not have good evidence.**
